# *Absence of parvalbumin increases mitochondria volume and branching of dendrites in inhibitory Pvalb neurons in vivo: a point of convergence of autism spectrum disorder (ASD) risk gene phenotypes*

**DOI:** 10.1186/s13229-020-00323-8

**Published:** 2020-06-09

**Authors:** Lucia Janickova, Karin Farah Rechberger, Lucas Wey, Beat Schwaller

**Affiliations:** Anatomy, Section of Medicine, University of Fribourg, Route Albert-Gockel 1, 1700 Fribourg, Switzerland

**Keywords:** Parvalbumin, Calcium-binding protein, Mitochondria, Calcium homeostasis, Pvalb neurons, Autism spectrum disorder

## Abstract

**Background:**

In fast firing, parvalbumin (PV)-expressing (Pvalb) interneurons, PV acts as an intracellular Ca^2+^ signal modulator with slow-onset kinetics. In Purkinje cells of PV^−/−^ mice, adaptive/homeostatic mechanisms lead to an increase in mitochondria, organelles equally capable of delayed Ca^2+^ sequestering/buffering. An inverse regulation of PV and mitochondria likewise operates in cell model systems in vitro including myotubes, epithelial cells, and oligodendrocyte-like cells overexpressing PV. Whether such opposite regulation pertains to all Pvalb neurons is currently unknown. In oligodendrocyte-like cells, PV additionally decreases growth and branching of processes in a cell-autonomous manner.

**Methods:**

The in vivo effects of absence of PV were investigated in inhibitory Pvalb neurons expressing EGFP, present in the somatosensory and medial prefrontal cortex, striatum, thalamic reticular nucleus, hippocampal regions DG, CA3, and CA1 and cerebellum of mice either wild-type or knockout (PV^−/−^) for the *Pvalb* gene. Changes in Pvalb neuron morphology and PV concentrations were determined using immunofluorescence, followed by 3D-reconstruction and quantitative image analyses.

**Results:**

PV deficiency led to an increase in mitochondria volume and density in the soma; the magnitude of the effect was positively correlated with the estimated PV concentrations in the various Pvalb neuron subpopulations in wild-type neurons. The increase in dendrite length and branching, as well as thickness of proximal dendrites of selected PV^−/−^ Pvalb neurons is likely the result of the observed increased density and length of mitochondria in these PV^−/−^ Pvalb neuron dendrites. The increased branching and soma size directly linked to the absence of PV is assumed to contribute to the increased volume of the neocortex present in juvenile PV^−/−^ mice. The extended dendritic branching is in line with the hypothesis of local hyperconnectivity in autism spectrum disorder (ASD) and ASD mouse models including PV^−/−^ mice, which display all ASD core symptoms and several comorbidities including cortical macrocephaly at juvenile age.

**Conclusion:**

PV is involved in most proposed mechanisms implicated in ASD etiology: alterations in Ca^2+^ signaling affecting E/I balance, changes in mitochondria structure/function, and increased dendritic length and branching, possibly resulting in local hyperconnectivity, all in a likely cell autonomous way.

## Background

Neurodevelopment involves many precisely regulated and temporally well-orchestrated mechanisms, finally leading to such a highly complex structure as the mammalian brain. It involves cell proliferation, complex patterns of migration to the expected location, and finally morphological and functional integration into the cellular network consisting of various cell types (neurons, glia, endothelial cells). Dysfunction of any of these processes leads to neurodevelopmental disorders including attention deficit hyperactivity disorder, childhood epilepsy, intellectual disability, and autism spectrum disorder (ASD). A close relationship between these disorders and interneuron dysfunction has been elaborated before [1]. During brain development, GABAergic signaling plays an essential role and not surprisingly, disturbances/disruptions of GABAergic transmission lead to excitation/inhibition (E/I) imbalance related to the pathogenesis of ASD. In particular, a subpopulation of GABAergic interneurons expressing the calcium-binding protein parvalbumin (PV; *Pvalb*), hereafter called Pvalb neurons, is in the center of focus in ASD research [2–4]. In the cortex of mice, Pvalb neurons represent the largest interneuron population (~ 40–50% of all interneurons [5]) and are characterized by their fast, non-adaptive firing properties, thus often called fast-spiking interneurons. Also, in human cortex Pvalb neurons are widespread to various extents; e.g., in the prefrontal cortex the percentage of PV^+^ chandelier and basket cells ranges from 25 to 50% depending on prefrontal areas [6]. Their targeting of the soma or the axon initial segments of excitatory neurons makes them particularly suitable to control the firing of pyramidal cells and synchronization of neuron ensembles [7, 8]. Pvalb neurons are present in various brain regions including neocortex, hippocampus, striatum, nucleus accumbens, thalamic reticular nucleus (TRN), globus pallidus, cerebellum, and olfactory bulb, just to name a few (for a detailed description of the distribution of Pvalb neurons in the rodent brain, see [9, 10]). Hence, PV immunoreactivity is widely used as a marker for these specific neuron subpopulations in different brain regions [11, 12]. While dysfunction of Pvalb neurons is undisputedly linked to neurodevelopmental [2–4] and also neuropsychiatric disorders (e.g., schizophrenia [13]), the putative role of the protein PV lending its name to the Pvalb neuron subpopulation has been investigated to a much lesser extent in these disorders.

PV (M_r_ 12 kDa) belongs to the family of EF-hand Ca^2+^-binding proteins (CaBPs) and acts as a cytosolic Ca^2+^-signal modulator (also termed Ca^2+^ buffer). Its particular property in physiological conditions consists of high affinity for Ca^2+^ (K_D_ 150–250 nM), but slow-onset kinetics of Ca^2+^ binding that makes PV unique among the identified ~ 250 EF-hand CaBPs in mice [14] (for a detailed review, see [15]). PV together with other proteins and organelles implicated in the regulation and modulation of the intracellular Ca^2+^ concentration ([Ca^2+^]_i_) is a component of the Ca^2+^-signaling toolkit [16]. The absence of PV in knockout mice (PV^−/−^) alters the kinetics of intracellular Ca^2+^ signals modifying short-term synaptic plasticity between cerebellar molecular layer Pvalb neurons and Purkinje cells [17], as well as at hippocampal [18] and striatal [19] Pvalb neuron synapses. At the morphological level, in all investigated PV^+^ cells including Purkinje cells, fast-twitch muscle and kidney epithelial cells absence of PV induces an upregulation of mitochondria, thought to represent a homeostatic/adaptive mechanism of the Ca^2+^-signaling toolkit (for details, see [15]). At the behavioral level PV^−/−^ mice show an evident ASD-like phenotype comprising all core symptoms and several comorbidities, as well as neuro-morphological abnormalities assumed to affect the E/I balance [20]. Of note, alterations/dysfunction of mitochondria is observed in several genetic mouse models of ASD and is also detected in postmortem brains of ASD patients (reviewed in [21]). Such alterations include changes in the levels or function of electron transport chain (ETC) complexes, oxidative stress, and mtDNA mutations. Moreover, a decrease in *PVALB* mRNA is one of the most conspicuous and consistent findings in human ASD brain samples [22, 23], and at the cellular level, e.g., in Purkinje cells [24]. Based on the close inverse relationship between PV expression and mitochondria volume, as well as their common impairment/dysfunction in ASD, we investigated the relative mitochondria volume in various Pvalb neuron populations in mice with or without PV. As mitochondria are also strongly linked to neuron morphology, in particular to growth and branching of processes [25], we examined the morphology of selected Pvalb neuron populations in PV-deficient mice. Our results indicate that in the absence of PV, not only the mitochondria volume is increased, but also the dendritic tree is larger and/or more complex indicative of local hyperconnectivity in these mice.

## Methods and materials

### Aim

Absence of PV in PV^−/−^ mice results in an upregulation of mitochondria volume in cerebellar Purkinje cells, while PV overexpression in several in vitro cell models causes a decrease in mitochondria volume indicative of an inverse, likely antagonistic regulation of PV and mitochondria. Whether mitochondria upregulation in PV^−/−^ mice is unique to Purkinje cells or common to all Pvalb neurons in various brain regions of PV^−/−^ mice is currently unknown, as well as whether the extent of mitochondria upregulation is correlated with PV expression levels in Pvalb neurons of wildtype (WT) mice. Thus, our first aim was to determine quantitatively morphological cell parameters (volumes of somata, nuclei, cytoplasm, mitochondria) of different WT and PV^−/−^ Pvalb neuron subpopulations in distinct brain regions including cortex, striatum, hippocampus, TRN, and cerebellum. We then attempted to correlate expected changes in any of these parameters (with a focus on mitochondria volume changes) with the PV concentration present in the various WT Pvalb neurons.

### Design

Transgenic mice selectively expressing the enhanced green fluorescence protein (EGFP) in Pvalb neurons allow for the identification of Pvalb neurons, irrespective of PV protein expression levels. Brain sections of PV-EGFP (WT) and PVKO-EGFP (KO) mice were stained with specific antibodies in order to visualize mitochondria in the soma and dendrites of selected Pvalb neurons characterized by high and low levels of PV expression. Identification of particular brain areas, characterized by distinct Pvalb neuron populations, was done with the help of the Allen brain atlas.

### Setting of the study

Initial characterization of PV levels in Pvalb neurons was carried out on whole-brain sagittal sections, where all Pvalb neuron subpopulations were present on a single section. Coronal brain sections were scanned at the level of the somatosensory cortex (SSC), medial prefrontal cortex (mPFC), hippocampus (CA1, CA3, DG), striatum, cerebellum, and TRN using confocal microscopy along the *z*-axis at 0.42 μm steps, with constant acquisition parameters for WT and PV^−/−^ mice. Morphometric analysis and 3D reconstruction of z-stack images was done in the Imaris 9.3.1 software. Western blot analysis was used to determine PV, EGFP, and GAPDH (control) protein levels.

### Animals

All experiments involving animals were performed according to the present Swiss law and the European Communities Council Directive of 24 November 1986 (86/609/EEC), with the permission of the local animal care committee (Canton of Fribourg, Switzerland); the authorization number for housing is H-04.2012-Fr and for experiments 2016_37_FR. Mice were group-housed at the University of Fribourg, Switzerland in a temperature-controlled animal facility (24 °C, 12:12 h light/dark cycle) and fed ad libitum. Only males from two transgenic lines, 3–5 months old, were used in this study. In the control (WT for the *Pvalb* gene) line B6.Tg (Pvalb-EGFP)^1Hmon^ [26] EGFP is expressed in Pvalb neurons and mice express normal levels of PV, while in the line B6.Pvalb^tm1Swal^ x B6Tg(Pvalb-EGFP)^1Hmon^ [19], in addition to EGFP expression in Pvalb neurons, endogenous PV expression is completely absent due to homologous deletion of both functional *Pvalb* alleles.

### Tissue preparation

Mice were anesthetized with 300 mg/kg body weight Esconarkon® (Streuli Pharma AG, Switzerland) and perfused using 0.9% NaCl, followed by perfusion with 4% PFA in 0.9% NaCl. Brains were removed, post-fixed for 24 h in 4% PFA in TBS and cryopreserved in 30% sucrose-TBS 0.1 M, pH 7.3 at 4 °C, as described before [27]. For RT-qPCR and western blot analysis, anesthetized mice were euthanized by exsanguination; brains were quickly removed and cut into half, along the fissure *longitudinalis cerebri*. Cortex and cerebellum were dissected as previously described [27] and snap-frozen in liquid nitrogen and stored at − 80 °C until further use.

### Immunohistochemistry (IHC)

Coronal and sagittal sections were cut into 40-μm sections using a freezing microtome (Leica SM2010R, Switzerland) as described before [27]. Briefly, free-floating sections were initially blocked with a solution containing 0.1 M TBS, 0.4% Triton X-100, and 10% Newborn Calf Serum (NCS) during 1 h at room temperature. Next, sections were washed three times with TBS, for 5 min each. Incubation with primary antibodies was performed overnight at 4 °C. As primary antibodies, the PV antibody (guinea pig anti-PV690, product code GP-72, Swant, Marly, Switzerland; 1:1000 dilution), the EGFP antibody (rabbit anti-EGFP, product code A6455, Molecular Probes, Thermo Fisher Scientific, Switzerland; 1:2000 dilution), and the COX I antibody (mouse monoclonal anti-cytochrome oxidase I (COX I), clone COX 111, product code #35-810, Molecular Probes, Invitrogen AG, Switzerland; 1:1000 dilution) were used. Sections were rinsed twice with TBS and once with Tris-HCl 0.1 M, pH 8.2, 5 min each. The following secondary antibodies were used: anti-guinea pig Cy3-conjugated antibody (Milan Analytic AG, Switzerland; 1:400 dilution), anti-rabbit Alexa488-conjugated antibody (Life Technologies, Thermo Fisher Scientific, Switzerland; 1:500), and anti-mouse Alexa647-conjugated antibody (Life Technologies, Thermo Fisher Scientific, Switzerland, 1:500). Sections were incubated with secondary antibodies for 1 h at room temperature. Finally, all sections were washed three times with TBS and nuclei were stained with DAPI (LuBio Science GmbH, Switzerland; 1:1000 dilution), for 5 min in TBS. After final rinsing, slides were transferred to MENZEL-GLÄSER SUPERFROST® (Thermo Fisher Scientific, Switzerland) and coverslipped with hydromount (National Diagnostics, Atlanta, Georgia, USA).

### Whole slide scanning

The sagittal brain sections were scanned by a whole digital slide-scanner Nanozoomer 2.0-HT (Hamamatsu Photonics K. K, Switzerland) or a wide-field upright microscope LeicaDM6B-Z Navigator (Leica Microsystems Inc., Buffalo Grove, IL) with HC PL APO20x, 0.80 NA dry objective, high speed sCMOS camera DFC9000GT-VSC07931, and fully automated stage and light path configurations. Identical parameters for scanning were applied for parallel-processed and stained sections of both (PV-EGFP and PVKO-EGFP) lines. Scanned images were viewed with the NDP.view2 software to identify specific staining for DAPI, Alexa488-, or Cy3-conjugated antibodies. In parallel sections, Cresyl violet staining was performed for quantitative anatomical comparison of brain sagittal sections. Apparent surface was then analyzed using the “free-hand” tool in the NDP.view2 software.

### Confocal microscopy

A laser scanning confocal microscope Leica TCS SP5 (Leica Microsystems Inc., Buffalo Grove, IL) equipped with motorized conventional Galvo stage was used in this study. Optical sections were acquired along the *z*-axis at 0.42 μm steps using a 40 × oil-immersion APO plan objective with 1.3 numerical aperture. The parameters of acquisition were the following: image format 1024 × 1024 pixels, 200 Hz scan speed, and the pinhole diameter was set to 1 AU to obtain a small, but very focused extract of the picture as described before [28]. All coronal brain sections were scanned at the SSC, mPFC, hippocampus (CA1, CA3, DG regions), striatum, cerebellum, and TRN.

### 3D-reconstruction and morphometric analysis of somata and dendrites

For image reconstruction and morphometric analyses, the complete series of z-stack images from the confocal microscope were processed using the Imaris 9.3.1® software (Bitplane, AG, Switzerland). The volume (V) of the soma cytoplasm (green), nucleus (blue), and mitochondria (red) were calculated for each neuron with the same parameters and algorithm settings using the “ImarisSurface” software package. Cells located at the border of the z-stack series (somata not entirely within the limits of the z-stack) were excluded from analysis. Each neuron fulfilling the above criteria was reconstructed by segmenting regions of interest. The source channel 1 (blue) was smoothened by “0.3-μm surface detail” and a threshold of 20 was selected based on the absolute intensity. For all channels, the filter type “number of voxel = 1” was used. For source channel 2 (green), surface details were smoothened by 0.4 μm and a threshold of 30 was selected based on absolute intensity. Source channel 3 (red) was smoothened by “0.1-μm surface detail” and a threshold of 50 was selected. Statistical analyses were performed using the “Imaris Measurement Pro” software package as described previously [28]. From each selected brain area of PV-EGFP and PVKO-EGFP mice, at least 50 neurons from 5 mice (10–15 cells/mouse) were analyzed. All values obtained by three different observers (blinded to the mouse genotype) were averaged resulting in one value per mouse per brain region (Pvalb neuron subtype). Results are presented in the graphs as one symbol per one mouse.

For automatic neuron tracing and analysis of neuronal branching, the “Filament Tracer” software package was used. Autopath method with “Trees without loops” algorithm was used for tracing filamentous structures in 3D. For the analyses of the images, obtained from 5 mice per genotype, 3 sections (details on the selection of the sections are described in “Image quantification”) representing the analyzed brain regions (DG, striatum, cerebellum (MLI)) and 3 neurons per section were analyzed. This made a total of 45 neurons per genotype and brain region as reported in Fig. 6 c, f, and i. Selection criteria were aimed to analyze neurons with a maximal dendritic tree in the *x*-*y* plane minimally overlapping with dendrites of adjacent neurons (e.g., shown in Fig. 6a). Neurons fulfilling the criteria were rather easily detected in the striatum, but more difficult to find in DG and cerebellum due to the relatively high density of Pvalb neurons (Fig. 6a, d, g, see also supplemental movie M1). Pvalb neurons from WT and KO mice were reconstructed automatically using the same algorithm settings and applying the following criteria: starting points (largest diameter, i.e., dendrite beginning) were detected by setting this value to 20 μm (representing the soma); seed points (thinnest diameter, i.e., dendrite ending) were defined by setting this value to 1 μm; and the threshold was selected automatically and disconnected segments were removed. The maximal gap length algorithm was set to 20 μm. For the dendrite diameter, the “shortest distance from distance map” algorithm was used. In order to eliminate neuropil (mostly axons) from the DG granule cell layer, seed points around a 150-μm-sphere diameter were removed. Besides reducing observer-linked bias, this automated analysis method was considerably faster than any manual tracing method.

The proximal and distal segments of dendrites from a given neuron were analyzed separately. For each analyzed dendrite segment, the cytoplasm signal (green) needed to be present in all confocal planes of the z-stack along the entire length of the segment of 20–30 μm. Thus, dendrites oriented diagonally within the z-stack (40 μm) were excluded, since the lateral resolution (*z*-*x* and *z*-*y*) is lower compared to the planar resolution (*x*-*y*) and would have led to imprecise measurements. From the 45 neurons per genotype and brain region used for reconstruction of the soma, 10 neurons (2 per mouse) from each analyzed brain region fulfilling the criteria were randomly selected for the analysis shown in Fig. 7. Proximal dendrites were analyzed starting from the soma, following the dendrite of the 1st branch order up to a distance of 20–30 μm. Distal dendrites were analyzed within 4th order branches, also along a total distance of 20–30 μm. In order to eliminate fluorescence signals from non-analyzed areas, a surface mask was applied and all signals outside the masked volume were discarded. The “surface channel” of the reconstructed dendrite was then changed to “Transparency mode,” which allowed to visualize mitochondria within the highlighted dendrite. Mitochondria localization within the dendritic segments was confirmed by rotation of each dendrite in all possible directions (360°) (see also supplemental movie M2 for better visualization of the selection process).

### Image quantification

For each mouse, coronal brain sections were collected following stereological systematic random sampling principles, providing accurate, unbiased, and quantitative estimates of objects within neuroanatomical structures [29, 30]. The different brain regions were defined (from bregma) as follows: mPFC from 1.87 to 2.22 mm, striatum from 0.38 to 0.62 mm, SSC, TRN, and hippocampus from − 1.94 to − 1.70 mm, and cerebellum (PC and MLI) from − 6.00 to − 5.76 mm. Stereotactic coordinates were confirmed by Allen brain atlas (http://atlas.brain-map.org/) and Paxinos Franklin atlas [31]. Representative sections localized in the middle of the different bregma zones are shown in suppl. Fig. S1 (e.g., + 2.10 mm for mPFC). If the middle section was the *n*th parallel section, then two additional ones, i.e., (*n* − 3)th and (*n* + 3)th were taken (e.g., sections 4, 7, 10; 7 representing the middle section). The identical procedure was applied for the selection of sections from WT and KO mice. A side-by-side comparison of the Nanozoomer images (brightfield mode and fluorescent mode, suppl. Fig. S1A & B, respectively) was performed to ascertain the presence of the same neuroanatomical structures on the 3 sections of both genotypes. The same sections were then scanned with the confocal laser-scanning microscope in 3D (z-stack) in regions of interest (white squares of 390 × 390 × 40 μm in suppl. Fig. S1B) according to *x*-*y* coordinates from the Nanozoomer, and thus matching (nearly identical) regions selected on sections from WT and KO brains. On some sections, only one brain area was analyzed (mPFC, striatum, cerebellum), while in others, several regions were analyzed in parallel (SSC, hippocampus (DG, CA3, CA1), TRN). Higher magnification images of the white-square regions are shown in suppl. Fig. S1C. Within a frame all Pvalb neurons, identified by EGFP immunolabeling, were selected from the entire z-stack of images, if they fulfilled the selection criteria. The complete soma needed to be contained in the z-scan, i.e., neurons cropped in the vertical (along the *z*-axis) or horizontal (*x*-*y*) planes were excluded from the quantification process. The remaining neurons were numbered randomly from 1 to *n*, *n* most often ranging from 3 to 9 per frame depending on the brain area; examples are shown in suppl. Fig. S1C. In regions densely packed by Pvalb neurons (TRN, cerebellum), neurons with EGFP^+^ neuropil from neighboring neurons in close apposition (complicating the precise delineation of the soma boundary) were also excluded from analyses. On the 3 sections and representing one brain area, on average 10–15 neurons were analyzed per mouse brain. Each neuron, fulfilling the criteria for analysis was marked (highlighted) throughout the entire z-stack, and then subjected to quantitative analyses by three independent observers, blinded to the mouse genotype (see also supplemental movie M3). Values for each neuron were first averaged using the results of the three observers. Then, values from all analyzed neurons in a given brain region (e.g., TRN) were averaged per region yielding one value per mouse and genotype. For the analyses of the branching and mitochondria density, the entire analyzed dendritic region had to be contained in the z-stack, in an orientation parallel to the *x*-*y* plane, as shown in the supplemental movie M2.

### Western blot analysis

Frozen brain tissue (cerebellum) of PV-EGFP and PVKO-EGFP mice was homogenized using a sonicator and resuspended in radio-immunoprecipitation assay RIPA-buffer supplemented with a Protease Inhibitor Cocktail (Thermo Fisher Scientific, Switzerland), then centrifuged at 12,000 rpm at 4 °C for 20 min, as described before [32]. The bicinchoninic acid BC Assay Protein Quantitation Kit (Uptima, Interchim, Switzerland) was used to determine the protein concentration of the samples. Denatured proteins (30 μg) were loaded on the gel, separated by SDS-PAGE and transferred onto nitrocellulose membranes (Trans-Blot® Turbo™ Transfer System, BioRad). The amount of proteins per lane was checked by Ponceau Red staining of the membrane after protein transfer. Next, the membrane was blocked with 5% milk for 1 h at room temperature and incubated with primary antibodies, rabbit anti-PV-25 (Swant, Marly, Switzerland; 1:10,000), rabbit anti-GFP (Thermo-Fisher Scientific, Switzerland; 1:10,000), and rabbit anti-GAPDH (Sigma-Aldrich, Buchs, Switzerland; 1:10,000) overnight at 4 °C. The membrane was washed three times and incubated with peroxidase-conjugated secondary antibody anti-rabbit (1:10,000 dilution, Sigma-Aldrich, Buchs, Switzerland) for 1 h at room temperature. The membrane was rinsed and developed using the FluorChem M System (ProteinSimple, Switzerland), as described previously [27].

### Statistical analysis

GraphPad Prism 7.04 software was used for statistical analysis. An unpaired, two-tailed *t* test was performed in order to compare the volumes and ratios between PV-EGFP and PVKO-EGFP neurons. For all experiments a *p* value < 0.05 was considered as statistically significant. Values are expressed as mean ± SD.

## Results

### Expression patterns of PV and EGFP in the transgenic mouse lines used in this study

**Fig. 1:**
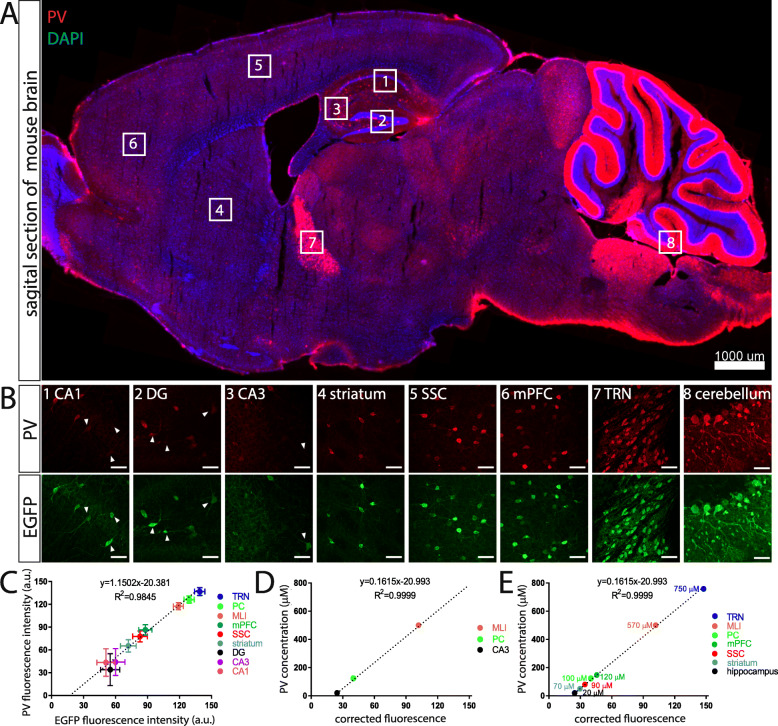
PV expression in 3–5 month old PV-EGFP mice. **a** Sagittal section showing PV^+^ Pvalb neurons (red) in various brain regions of a PV-EGFP mouse. Sections are co-stained with DAPI (blue) to identify cell nuclei. The analyzed regions 1–8 obtained by microscopy are shown at higher magnification and were obtained by confocal microscopy in (**b**); scale bar in (**a**) 1000 μm. **b** Confocal images of Pvalb neurons of a PV-EGFP mouse in the regions 1–8 stained for PV (upper row) and for EGFP (lower row), scale bar 50 μm. **c** Strong correlation between green EGFP signals (in a.u.) and red PV signals (in a.u.) in the 8 investigated regions indicates that EGFP signals are a reliable proxy measure of PV concentrations. Note the larger SD values for the PV staining than for EGFP staining in low-PV expressing Pvalb neurons (DG, CA3, CA1). **d** Representative standard curve using data from (**c**) of (corrected) EGFP signals vs. PV concentrations previously reported in (from low to high) hippocampal Pvalb neurons, PC, and cerebellar basket cells. **e** Estimation of PV concentrations in other Pvalb neuron subpopulations (for details, see suppl. material)

With the aim to investigate the effects of PV on the morphology of various Pvalb neuron subpopulations in different brain regions of adult mice (3–5 months) in vivo, we made use of two previously generated transgenic mouse lines [19, 26]. The genome of mice of the line B6.Tg (Pvalb-EGFP)^1Hmon^ (short: PV-EGFP or WT) contains a BAC transgene comprising the mouse *Pvalb* promoter driving EGFP, resulting in EGFP expression in a large majority of Pvalb neurons throughout the brain allowing to identify these neurons by their intrinsic green fluorescence [26]. The second line B6.Pvalb^tm1Swal^ x B6.Tg (Pvalb-EGFP)^1Hmon^ (short: PVKO-EGFP or KO) also expresses EGFP in Pvalb neurons, however lacks expression of PV, since they derive from a crossing with mice that are homozygous for the deletion of the functional *Pvalb* gene (B6.Pvalb^tm1Swal^ [33]). Although both strains were previously used to detect PV-mediated alterations in the electrophysiological properties of striatal Pvalb neurons [19] and PV-EGFP mice additionally for determining the number of Pvalb neurons in different brain regions [30], a thorough morphological comparison between Pvalb neurons with or without PV expression was lacking. In sagittal sections of PV-EGFP mice, PV IHC revealed the presence of Pvalb neurons in different brain regions including medial prefrontal cortex (mPFC), somatosensory cortex (SSC), hippocampus (CA1, CA3, dentate gyrus (DG)), striatum, TRN, and cerebellum (Fig. 1a). Higher magnification confocal images of representative Pvalb neurons in the 8 selected regions are shown in Fig. 1b, (upper). In line with previous reports [9, 10], the size of the Pvalb neuron somata as well as the density of Pvalb neurons differed among brain regions and also the staining intensity (reflecting PV concentration) of Pvalb neurons varied both between Pvalb neuron populations from different brain regions, as well as between Pvalb neurons within a given region, as exemplified in cortical regions (SSC, mPFC; Fig. 1b). Pvalb neurons with rather low- or medium-intensity signals were prevailing in the hippocampus and in cortical regions, respectively, while much stronger PV immunofluorescence signals were observed in TRN and cerebellum, in the latter in both Purkinje cells and MLI. Staining of the same sections for EGFP yielded an almost identical pattern demonstrating expression of EGFP selectively in Pvalb neurons (Fig. 1b; lower part). While in strongly (red) stained Pvalb neurons, the overlap with EGFP signals was 100%, the hippocampal Pvalb neurons with faint-to-none PV staining were clearly positive for EGFP (marked by arrowheads) indicative of lower sensitivity of PV staining resulting in neurons, where the PV signal likely fell below the threshold for IHC. Based on the rather similar relative fluorescence signal intensities of Pvalb neurons either stained for intrinsic PV or the *Pvalb* promoter-driven EGFP (compare upper and lower parts of Fig. 1b), we established a curve plotting averaged green vs. averaged red fluorescence intensities (in a.u.) for various Pvalb subpopulations, which resulted in a near-linear relationship (Fig. 1c); since absolute a.u. values for PV and EGFP signals varied between sections and animals, representative values obtained in the various brain regions of one mouse are shown (Fig. 1c). Thus, we used the more robust EGFP intensities (generally seen by the smaller SD values, especially apparent in low-PV expressing hippocampal neurons) to estimate the PV concentrations in a given Pvalb neuron subpopulation. Since the absolute (not relative) concentrations of PV have been determined or estimated before in only a limited set of Pvalb neuron populations, i.e., mouse hippocampal interneurons (average 11.9 ± 1.6  µM, range 0.8–70.6  µM) and cerebellar basket cells (average 563 ± 66  µM; range 55–1788  µM, [34]), as well as rat Purkinje cells (~ 100  µM [35]), we used EGFP signal data (corrected for neuron morphology; for more details on the quantification, see suppl. material) to generate a standard curve (Fig. 1d) and then to roughly estimate hitherto unknown PV concentrations of Pvalb neuron subpopulations (Fig. 1e). Of note for hippocampal neurons, we chose a slightly higher (previously reported ~ 12  µM) value of 20  µM, since for the comparison of red and green fluorescent signals in hippocampal Pvalb neurons, we selected cells with both a distinct red (PV) and green (EGFP) signal and thus low-PV cells (green only) were under-represented in our analysis. This then allowed to roughly estimating PV concentrations of Pvalb neurons in striatum (~ 60–80  µM), cortical areas (SSC ~ 80–100  µM; mPFC ~ 110–140  µM), and TRN (~ 600–900  µM) (Fig. 1e and suppl. Table S1).

Next, we ascertained that the various subpopulations of Pvalb neurons were equally present in PV-deficient mice via their identification by EGFP expression. The distribution patterns of EGFP and PV expression in PV-EGFP and PVKO-EGFP mice were analyzed on sagittal sections by the Nanozoomer allowing to scan the entire sections at high resolution (suppl. Fig. S2A). As expected and in line with previous reports [30, 36], no PV signal (red) was detected in PVKO-EGFP mice, shown at higher magnification for Purkinje cells and MLI in suppl. Fig. S2B (left). On the contrary, staining for EGFP resulted in qualitatively indistinguishable images, when comparing to Nanozoomer-acquired images from whole brain sections of PV-EGFP and PVKO-EGFP mice (suppl. Fig. S2A). Also, images from the Purkinje cell layer and molecular layer of the cerebellum acquired by confocal microscopy were not different between the two mouse lines (suppl. Fig. S2B (middle)). Merged images from cerebellum showed complete overlap (yellow) in PV-EGFP mice confirming expression of EGFP selectively in all Pvalb neurons in this brain region. Western blot analyses with cerebellar tissue lysates confirmed IHC results, equally strong signals for EGFP (M_r_ 32 kDa) in both mouse lines and absence of PV (M_r_ 12 kDa) expression in PVKO-EGFP samples (suppl. Fig. S2C).

**Fig. 2:**
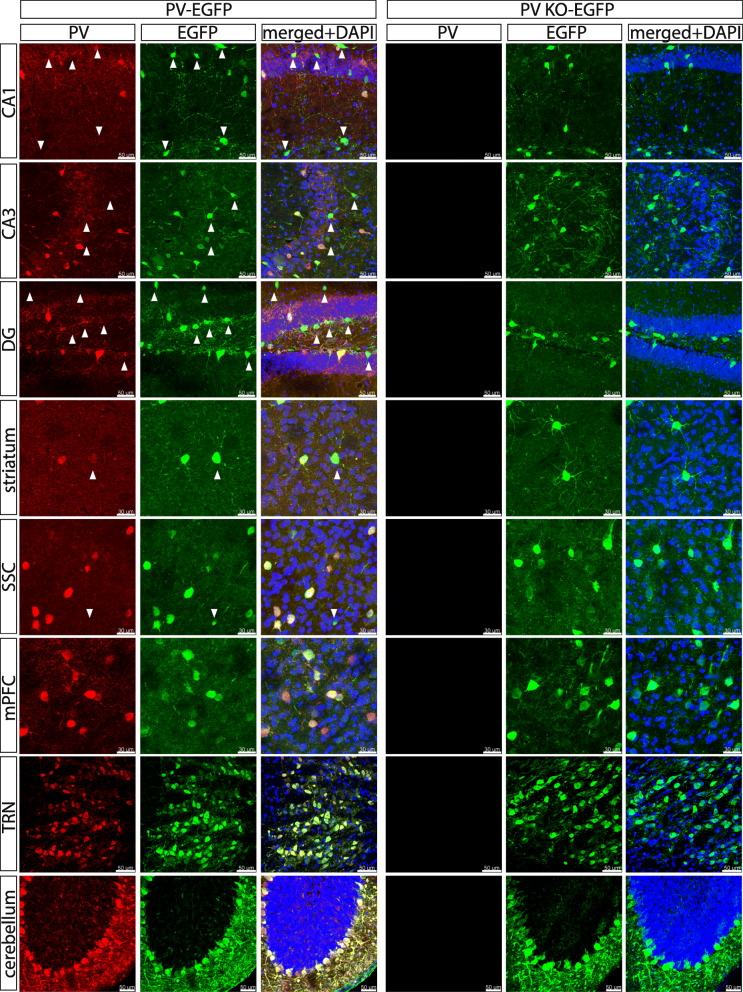
Co-staining for PV (red), EGFP (green), and DAPI (blue) in 8 different brain regions from PV-EGFP and PVKO-EGFP mice. In sections from PV-EGFP mice, note complete co-localization (yellow cells in the merged images) of PV and EGFP in regions with high PV expression levels (mPFC, SSC, striatum, TRN, and cerebellum). In all hippocampal regions (CA1, CA3, DG), a fraction of green (EGFP) cells show weak-to-absent PV staining (red) and green (only) cells (arrowheads) are seen in the merged images indicative of generally low PV expression levels in hippocampal Pvalb neurons. Yet the higher sensitivity for EGFP allows to identify those low-PV Pvalb neurons. The density and morphology of Pvalb cells (evidenced by EGFP staining) is qualitatively similar in all investigated regions in PV-EGFP (left) and PVKO-EGFP (right) mice. Scale bars: mPFC, SSC, and striatum 30 μm, CA1, CA3, DG, TRN, and cerebellum 50 μm

Co-localization of PV (red), EGFP (green), and DAPI (blue) signals was investigated in all 8 brain regions shown in Fig. 1. Evidently, no red signals (PV) were detected in PVKO-EGFP mice (Fig. 2, right panels). The overall cell soma morphology, distribution, and density of EGFP^+^ Pvalb neurons were qualitatively similar in mice with or without PV (compare EGFP signals in WT and KO mice; Fig. 2). In the investigated regions in WT mice, essentially all PV^+^ cells (red) displayed also EGFP (green) signals detected as yellow staining in the merged images. While a complete overlap between PV^+^ and EGFP^+^ cells was observed in Pvalb neurons known to express high PV levels (TRN, cerebellum), in cortical and most visibly in hippocampal regions, some of the EGFP^+^ cells showed sometimes weak red staining, at times barely above background staining, thus appearing mostly green in the merged images (Fig. 2). Some examples are marked by arrowheads. Since the average PV concentration in hippocampal Pvalb neurons (~ 12  µM) is approximately 50-times lower than in cerebellar basket cells (~ 570  µM) in mice [34], some weak hippocampal PV signals likely fell below the detection limit of IHC, possibly indicative of a lower sensitivity of the PV antibody compared to the EGFP antibody. A lesser degree of PV and EGFP colocalization in the hippocampus, i.e., a fraction of EGFP^+^ cells without detectable PV staining, had been reported in the initial characterization of the PV-EGFP mouse line [26].

### Macroscopic brain morphology of PV-EGFP and PVKO-EGFP mice

Since differences in brain volumes (neocortex, cerebellum) are present in PVKO mice at postnatal day 20 (PND20), but not in adult (> 12 months) mice [20], we carried out a basic macroscopic analysis of the apparent brain surface of 3–5-months old PV-EGFP and PVKO-EGFP mice used in this study, similarly as reported before [37]. Surface quantifications of brain structures were compared using dorsal macroscopic views (suppl. Fig. S2D). No significant differences were observed between brain surface areas of all investigated regions (olfactory bulbs, cortex, colliculi, cerebellum, whole brain) in the two genotypes. Cresyl violet-stained mid-sagittal sections yielding higher resolution images were used for the analysis of the apparent surface of hippocampus, cortex, striatum, cerebellum, and thalamus (suppl. Fig. S2E). None of the investigated regions were obviously different between the two strains. These results indicate that the absence of PV in the brain of 3–5-months old mice does not significantly affect brain gross anatomy, as is also seen in PV^−/−^ mice > 12 months [20].

### Effect of PV on the volumes of soma, cytoplasm, nucleus, and mitochondria of Pvalb neurons in vivo

**Fig. 3:**
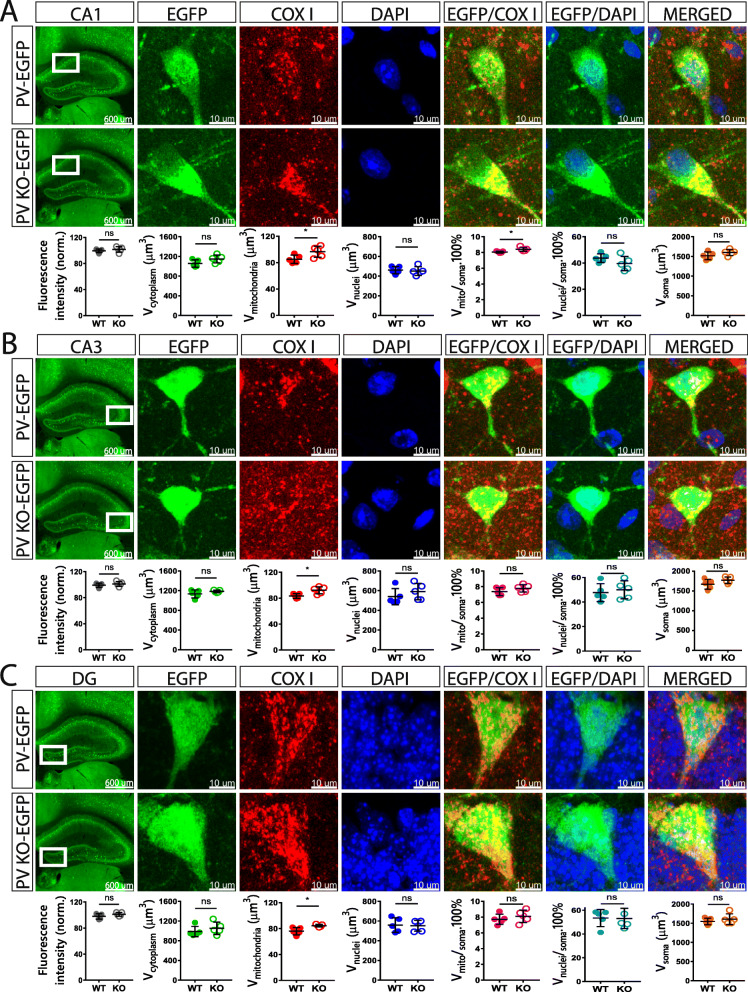
Quantitative morphological analyses of Pvalb neurons from PV-EGFP and PVKO-EGFP mice in the hippocampal regions CA1 (**a**), CA3 (**b**), and DG (**c**). Low magnification images of the analyzed brain regions are shown in the left panels (scale bars 600 μm). Higher magnification images (scale bar 10 μm) show representative Pvalb neurons stained for EGFP (green; cytoplasm), COX I (red; mitochondria), and DAPI (blue; nucleus). Partially merged images EGFP/COX I reveal mitochondria within Pvalb neurons, EGFP/DAPI images were used to distinguish cytoplasmic regions from nucleus and a merge of all three images is shown in the right. Overall fluorescence intensity (grey circles) measurements reveal similar global EGFP intensity stainings in sections from PV-EGFP and PVKO-EGFP mice. Analyzed parameters include the following: volume of cytoplasm (V_cytoplasm_; green), mitochondria (V_mitochondria_; red), and nuclei (V_nuclei_; blue) in a given Pvalb neuron. Additional parameters were calculated: ratio V_mitochondria_/V_soma_ (magenta), ratio V_nuclei_/V_soma_ (cyan), and soma volume (orange). Each dot in the graphs represents the average obtained in 1 animal (5 per genotype) and 10–15 cells per animal resulting in > 50 cells per brain region per genotype. ns not significant, *p < 0.05, **p < 0.01, ***p < 0.001, ****p < 0.0001

**Fig. 4:**
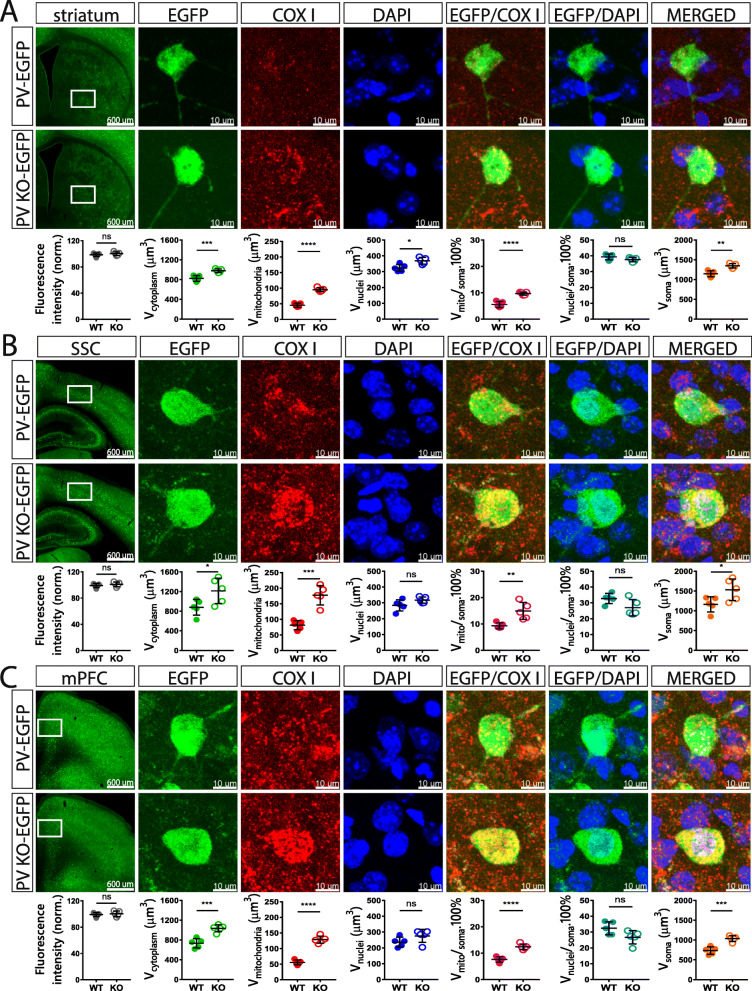
Quantitative morphological analyses of Pvalb neurons from PV-EGFP and PVKO-EGFP mice in striatum (**a**), SSC (**b**), and mPFC (**c**). The description and details are identical to Fig. 3. Scale bars for low magnification images of the analyzed brain regions shown in the left panels are 600 μm. Higher magnification images show representative Pvalb neurons with 10 μm scale bars

**Fig. 5:**
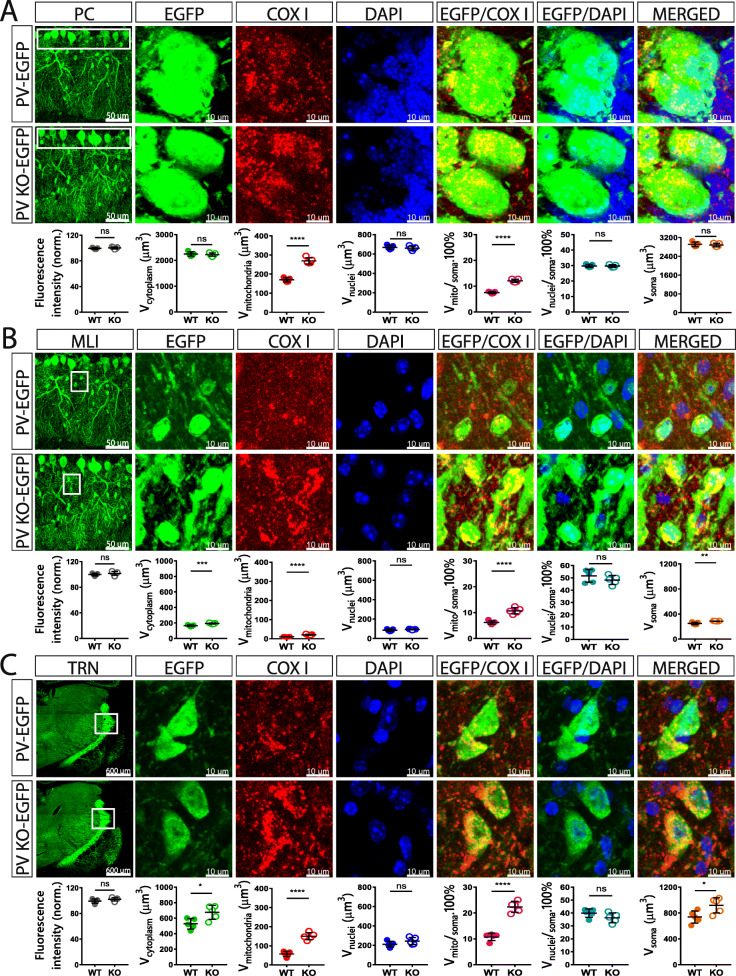
Quantitative morphological analyses of Pvalb neurons from PV-EGFP and PVKO-EGFP mice in the cerebellum: Purkinje cells (**a**), MLI (stellate and basket cells) (**b**), and Pvalb neurons in the thalamic reticular nucleus (**c**). The description and details are identical to Fig. 3. Scale bars for low magnification images of the analyzed brain regions shown in the left panels are 50 μm (PC, MLI) and 600 μm (TRN). Scale bars for higher magnification images are 10 μm

An inverse relationship between PV expression and mitochondria volume, i.e., a decrease in PV resulting in mitochondria upregulation, exists in all tissues/systems investigated so far: in fast-twitch muscles, kidney epithelial cells, and Purkinje cells of PV^−/−^ mice (for a review, see [15]). However, whether such a regulation in the brain was specific for Purkinje cells or also occurring in other Pvalb neuron subpopulations was hitherto unknown. Thus, confocal microscopy on coronal sections followed by 3D reconstruction was used to determine volumes of soma, cytoplasm, nucleus, and mitochondria of Pvalb neurons in the regions shown in Fig. 1. Furthermore, the ratio volume of mitochondria per volume of soma (V_mitochondria_/V_soma_), as well as the volume of nucleus per volume of soma (V_nucleus_/V_soma_) was calculated. For the determination of the somata volumes, sections were stained for EGFP, DAPI staining allowed to determine the volume of nuclei and COX I immunostaining was used to obtain volumes of mitochondria, as was used in our previous study [38]. DAPI staining also allowed to delimitate better specific brain regions (cortex, striatum, hippocampus, and cerebellum). All coronal brain sections were additionally identified by the Allen brain atlas and imaged with both, the Nanozoomer and confocal microscopy. Representative images of brain areas of our interest together with representative Pvalb neurons in the corresponding regions are shown in Figs. 3, 4, and 5. Based on the intensity of EGFP staining (a proxy measure of the PV concentrations as shown in Fig. 1c), regions of similar EGFP staining intensities were grouped together. Weak EGFP signals were detected in hippocampal regions *stratum oriens* of the CA1 region (Fig. 3a), *stratum oriens* of the CA3 region (Fig. 3b) and DG (Fig. 3c); intermediate-intensity EGFP signals were present in cortex and striatum (Fig. 4a–c), and brain regions with high-intensity EGFP signals consisted of cerebellum (Purkinje cells and MLI) and TRN (Fig. 5a–c). Of note, EGFP signal intensities were also compared between the same brain region-specific Pvalb neurons of PV-EGFP and PVKO-EGFP mice. The nearly identical values of EGFP signal intensities in both strains indicated that the activity of the *Pvalb* promoter was not visibly affected by the absence of PV in the PVKO-EGFP line. In PV-deficient mice, the absolute mitochondria volume was increased in Pvalb neurons in a graded manner: the largest increase (+ 161%) in TRN neurons expressing the highest PV levels (strongest EGFP staining), a rather similar increase in most other Pvalb neurons including striatal (+ 109%), SSC (+ 117%) and mPFC (+ 131%) Pvalb neurons, and MLI (+ 104%), a smaller one in Purkinje cells (+ 58%), and a minor increase in the order of maximally 10% in hippocampal (DG, CA3, CA1) Pvalb neurons (suppl. Fig. S3B). When comparing the increase in the mitochondria density, i.e., taking into account the increase in the cytoplasm volume caused by PV-deficiency, then the increase in MLI mitochondria density better correlated with the PV concentrations present in the various Pvalb neurons (suppl. Fig. S3C). The increase in mitochondria volume (density) is also reflected by a relatively similar, yet smaller increase in total soma volume (suppl. Fig. S3D). More importantly, the relative increase in the density of mitochondria caused by the absence of PV generally correlated with the prevailing (or estimated) PV concentrations in the corresponding Pvalb neurons in WT mice (Fig. 1). That is, the higher the PV concentration in a WT Pvalb neuron, the larger the increase in mitochondria density in absence of PV (suppl. Fig. S3C). As analogously observed before in PV-expressing CG4 cells in vitro, also the cytoplasmic volume, as well as the volume of the entire soma were increased in the absence of PV, and again was significant in Pvalb neurons with medium-to-high PV expression levels. Of importance, the relative mitochondria density was increased and is thus not merely the result of an increase in overall cytoplasmic volume. On the other hand, volumes of nuclei were only slightly—and in most cases not significantly—increased by the absence of PV (except in striatal Pvalb neurons + 14%) and moreover ratios V_nucleus_/V_soma_ were unchanged indicative of a proportionally similar increase of nuclei and cytoplasm in PV-deficient Pvalb neurons. All values obtained in individual mice, as well as all statistical analyses of the data are presented in Figs. 3, 4, and 5.

### Dendrite morphology of Pvalb neurons is affected by PV expression and correlated with dendrite mitochondria length and density

**Fig 6:**
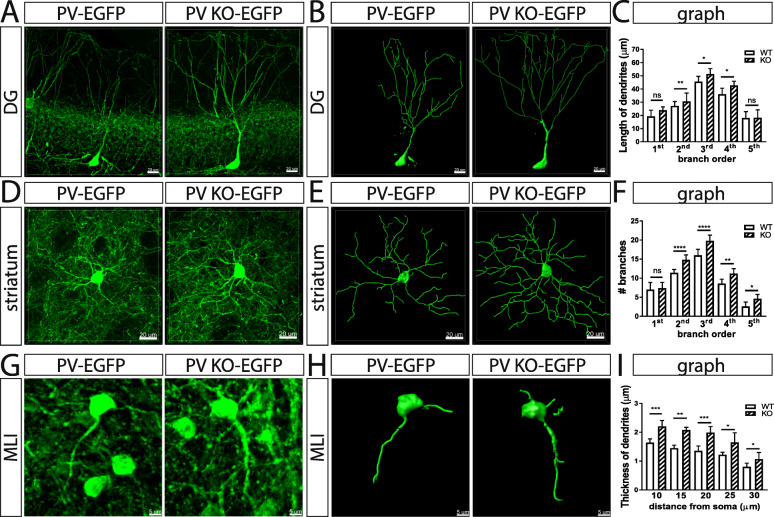
Morphological analyses of the dendritic tree of Pvalb neurons in the hippocampal DG (**a**–**c**), striatum (**d**–**f**), and MLI (**g**–**i**) of PV-EGFP and PVKO-EGFP mice. **a** Representative images (EGFP fluorescence) of a DG Pvalb neuron of both genotypes. **b** 3D-reconstruction of the same neurons using the Imaris software. **c** Quantitative assessment of number of branches on 1st–5th order dendrites. For all images: ns not significant, *p < 0.05, **p < 0.01, ***p < 0.001, ****p < 0.0001. **d** Representative images (EGFP fluorescence) of striatal Pvalb neurons of both genotypes. **e** 3D-reconstruction of the same neurons using the Imaris software. **f** Quantitative assessment of length of dendrites from 1st to 5th order branches. **g** Representative images (EGFP fluorescence) of MLI of both genotypes. **h** 3D-reconstruction of the same neurons using the Imaris software. **i** Quantitative assessment of thickness of primary dendrites at a distance of 10, 15, 20, 25, and 30 μm away from the soma

**Fig. 7:**
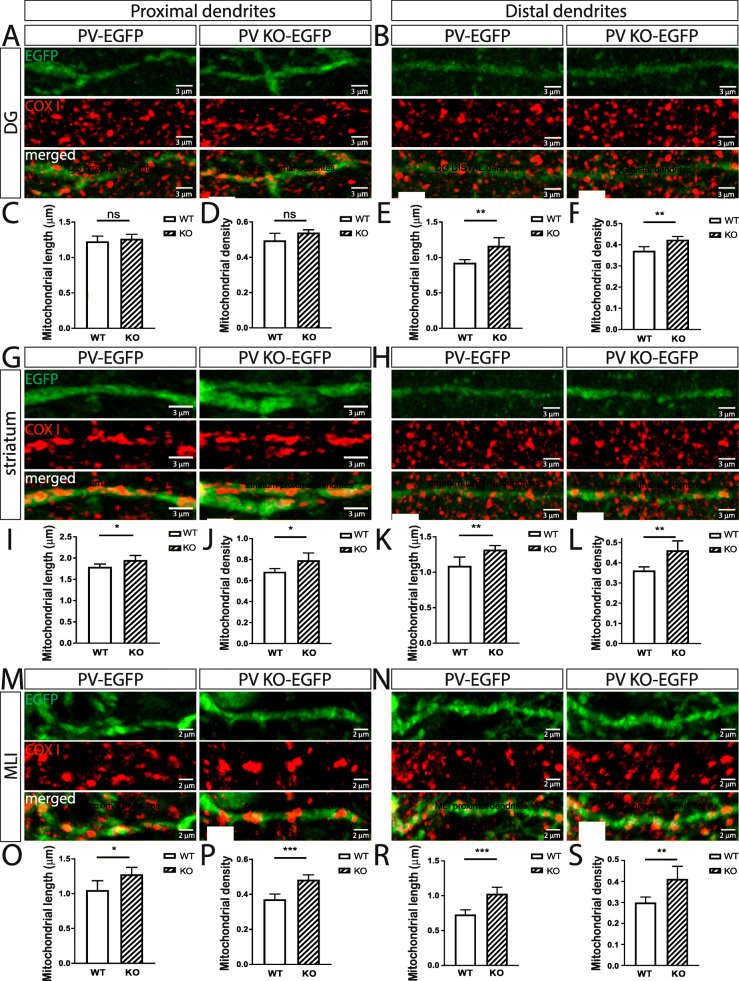
Mitochondria length and density in proximal and distal dendrites of hippocampal DG (**a**–**f**), striatal (**g**–**l**), and MLI (**m**–**s**) Pvalb neurons from PV-EGFP (WT) and PVKO-EGFP (KO) mice. **a** Representative images of proximal dendrites from DG Pvalb neurons of a PV-EGFP (left) and a PVKO-EGFP (right) mouse showing the overall dendrite morphology (EGFP, top), mitochondria (COX I, middle), and the merged image (bottom). **b** Images from distal dendrites (as in (**a**)). Average length and density of mitochondria in proximal (**c**, **d**) and distal (**e**, **f**) dendrites of DG Pvalb neurons. Representative images (**g**, **h**) and quantitative analyses (**i**-**l**) from striatal Pvalb neurons. Representative images (**m**, **n**) and quantitative analyses (**o**-**s**) from MLI Pvalb neurons. For all graphs showing quantitative data: *n* = 10 randomly selected cells and ns: not significant, *p < 0.05, **p < 0.01, ***p < 0.001

Since mitochondria are implicated in the formation of cell processes, in particular in branching of axons and dendrites in neurons [39, 40] and PV was previously shown to affect glial process formation in differentiated CG4 cells [28] and moreover to affect dendritic branching observed in PND18-24 striatal Pvalb neurons of PVKO mice [20], we were interested whether PV altered dendrite morphology in other Pvalb neurons as well. In a first step, we ascertained whether morphological changes of Pvalb striatal neurons seen in PND18-24 mice [20] also persisted in adult PVKO-EGFP mice of 3–5 months. Sholl analysis of striatal PVKO-EGFP Pvalb neurons was performed as before [20] and revealed that the number of 2nd to 5th order branches was significantly increased compared to PV-EGFP Pvalb neurons (Fig. 6d–f); similarly in PND18-24 PVKO-EGFP mice, a significant increase had been observed before for 3rd to 5th order branches (the increase in 2nd order branches just fell below significance) [20]. Although PV expression levels in Pvalb neurons in the hippocampus are relatively low and increase in soma mitochondria volume were small in absence of PV (Fig. 3), morphological changes were also observed in hippocampal PVKO-EGFP Pvalb neurons. The DG region was chosen for analysis due to the relatively low density of Pvalb neurons compared to the higher density in the CA1 and CA3 regions. This allowed to better visualizing the dendritic arbor of individual neurons. The length of 2nd to 4th order dendritic branches was clearly increased in PV-deficient Pvalb DG neurons (Fig. 6a–c) and thus the entire dendritic tree was visibly longer. The high density of Pvalb neurons resulting in overlapping dendritic trees precluded Sholl analyses of Pvalb neurons with high PV concentrations including TRN and cerebellar Pvalb neurons (Fig. 6g). However, quantitative morphological analyses revealed that the thickness of proximal dendrites of MLI Pvalb neurons (measured 10–30 μm away from the soma) was increased in the absence of PV (Fig. 6h, i). This indicates that absence of PV affects branching, as well as thickness of proximal dendrites, supporting previous studies [20]. Since decreased processes’ length and branching in PV-overexpressing oligodendrocyte-like CG4 cells in vitro is strongly correlated with reduced mitochondria density in these processes [28] and moreover mitochondria length is decreased in PV-overexpressing MDCK cells [41], we investigated these two parameters in proximal and distal dendrites of striatal and DG Pvalb neurons, as well as in MLI in PV-EGFP and PVKO-EGFP mice (Fig. 7). In line with the high variability of relative mitochondria density (7–42%) present in Purkinje cell distal dendrites of WT and PV^−/−^ mice reported before [42], a clearly larger variability compared to CG4 cells was also observed in the investigated Pvalb neurons. In low-PV expressing DG Pvalb neurons’ proximal dendrites, no differences in mitochondria length and density were observed in the absence of PV (Fig. 7a, c, d); however, both parameters were increased in distal dendrites of PV^−/−^ DG Pvalb neurons (Fig. 7b, e, f). In medium- and high-PV expressing striatal (Fig. 7g–l) and MLI (Fig. 7m–s) Pvalb neurons, respectively, in the absence of PV, mitochondria length and density were increased in the proximal, as well as in distal dendrites (Fig. 7). Of note, the relative increase in both parameters resulting from PV-deficiency was again highly correlated with the estimated PV concentration in the respective WT Pvalb neurons: a minor increase in DG Pvalb neurons, a larger one in striatal Pvalb neurons, and the biggest effect were notable in high-PV MLI Pvalb neurons (suppl. Fig. S4). The findings on PV-dependent changes in dendritic mitochondria density are in complete agreement with results obtained in vitro in processes of undifferentiated and differentiated CG4 cells with or without PV expression (see Fig. 6 in [28]).

## Discussion

PV-expressing neuron subpopulations are present throughout the mouse brain, yet the prototypical GABAergic, most often fast-spiking interneuron-type neurons are prevalent in particular regions including cortical, striatal, hippocampal (DG, CA3, CA1), and cerebellar regions, as well as TRN. Based on our semi-quantitative analyses of PV expression levels, MLI and TRN Pvalb neurons have the highest PV concentrations, the latter on average in the order of 600–900  µM. The most distinct function of PV with respect to intracellular Ca^2+^ signal modulation and thus distinguishing PV from essentially all other EF-hand CaBPs is the slow-onset of Ca^2+^ binding. The only other Ca^2+^ “off-mechanism” decreasing the intracellular Ca^2+^ concentration with similar slow kinetics is provided by mitochondria. This is the likely reason for the antagonistic regulation of PV and mitochondria volume pertaining in all investigated excitable and non-excitable systems/cell types so far: Purkinje cells, fast-twitch muscle, kidney epithelial cells (reviewed in [15]), and oligodendrocyte-like CG4 cells [28]. Although soma mitochondria volume is increased in Purkinje cells of PV^−/−^ mice [42], information on mitochondria volume changes in other PV-deficient Pvalb neuron populations was lacking. Here, we have demonstrated that absence of PV in PVKO-EGFP mice entailed an upregulation of mitochondria in the somata of all investigated Pvalb neurons. Importantly, the relative magnitude of the increase was highly correlated with the prevailing PV concentration in the respective Pvalb neurons in WT mice. This is indicative of a precisely regulated system, not a simple on/off mechanism. Another factor possibly affecting the magnitude of mitochondria upregulation caused by absence of PV might be linked to the activity-dependent gene expression (excitation-transcription coupling [43]) in Pvalb neurons, a cell population characterized by tremendous Ca^2+^ dynamics. Pvalb neurons make use of the particular gamma Ca^2+^/CaM-dependent protein kinase I (γCaMKI; *Camk1g*)-dependent pathway to trigger CREB phosphorylation and gene expression, e.g., of previously known activity-driven genes (*c-Fos*, *Arc*, *Wnt2*), but importantly also of *Gad1* (encoding GAD67) and *Pvalb* [44]. Thus, reduced activity of Pvalb neurons might contribute to PV downregulation and induce adaptive increase in mitochondria in these neurons. Of note, the *CAMK1G* transcript is also among the strongest downregulated ones detected in gene arrays from human ASD samples [23]. Not only the mitochondria volume was increased in absence of PV, but also the volume of the cytoplasm resulting in an overall increase of soma volume in the order of ~ 15–40% in the medium-to-high PV-expressing Pvalb neurons. Although a similar increase in soma size caused by reduced PV levels exists in CG4 glial and MDCK epithelial cells in vitro [28, 41], such an increase in cell volume of a neuron subpopulation in a tissue as densely packed by cells as the brain was unexpected. Possibly the rather low density of Pvalb neurons in most brain regions, in particular cortical and sub-cortical regions, allows for such an increase in the soma volume. The only exception is Purkinje cells, where the soma volume was not increased in absence of PV, although the increase in mitochondria volume (~ 60%) is in the same range as in the nearby stellate and basket cells. This might be linked to the particular arrangement of Purkinje cells, as they form a densely packed contorted 2D-like layer entirely covering the granule cell layer. Thus, an increase in soma volume size of 30–40% would evidently result in a reduced number of Purkinje cells on the available surface delimited by the granule cell layer. Otherwise, the large cytoplasmic volume of Purkinje cells might be sufficient to harbor the additional mitochondria without necessitating an increase in soma size.

### Pvalb neurons, mitochondria dysfunction, and ASD

Impaired function of Pvalb neurons has been reported in several neurodevelopmental (ASD) and neuropsychiatric (schizophrenia) disorders [1]. Numerous potential mechanisms generally implicated in ASD pathophysiology have been proposed including alterations of synaptic transmission resulting in E/I imbalance, impaired neuronal Ca^2+^ signaling, changes in network connectivity, and mitochondrial dysfunction, to name a few. The last is prevalent in a much higher proportion in children with ASD (~ 5%) than in controls (~ 0.01%) [45]. Making use of their negative membrane potential (ΔΨ_mito_ ~ − 180 mV), mitochondria are involved in the regulation of intracellular Ca^2+^ dynamics in neurons, as well as controlling ATP availability. Moreover, alterations in mitochondria dynamics encompassing biogenesis, fission and fusion, intracellular mobility, and mitophagy have been reported in ASD [45]. In children with ASD (ages 4–10), but not in adults (ages 14–39), brain-region specific decreases in ETC complexes were observed indicative of mitochondria dysfunction rather than mitochondria disease [46]. Fecher et al. [47]) have demonstrated that mitochondria are highly variable and dynamic structures with respect to composition (molecular diversity) and function. A substantial differential expression of ~ 15% of mitochondrial proteins in Purkinje cells, granule cells, and astrocytes in the cerebellum indicate a hitherto unappreciated cell-type specific regulation of even basic mitochondrial functions in the CNS [47]. In view of these results, it is problematic to draw concise conclusions from the results by Chauhan et al. [46], since nothing is currently known about the origin of the changes (glia, neurons, neuron subpopulations) in the abundance of ETC complexes. Also, the functional consequences of decreased ETC complexes in ASD are not straightforward. On the contrary, in PND56 VPA rat tissue from dorsal hippocampus, aberrant enzymatic activity of mitochondrial ETC complexes is seen without changes in protein levels in OXPHOS complexes I–V [48].

Data from transcriptomic studies (RNA-seq of post-mortem ASD cerebral cortex samples) indicate that overall mitochondria might be downregulated in ASD [23], conforming with results obtained in children with ASD [46]. The most strongly downregulated genes were related to synaptic transmission and mitochondria. In line, transcriptomic network analysis on 3 mouse ASD and schizophrenia models revealed two cortical (c) and two hippocampal (h) modules (M) of co-expressed genes dysregulated in all three models [49]. The downregulated cM2 is enriched for genes linked to mitochondrial related energy balance (“energy-coupled proton transport” and “respiratory electron transport chain”). Yet, expression weighted cell type enrichment analysis revealed a significant increase in mitochondria-associated cM2 genes (annotated name: “neuronal-associated mitochondrial”) in fast firing inhibitory neurons, presumably Pvalb neurons [49]. Interestingly, the upregulated cM1 module is enriched for genes that are linked to cell morphology (“morphogenesis of branching structures”; for more details, see below). Thus, this data is compatible with global mitochondria impairment/downregulation in ASD (not investigated in our study), yet with increased mitochondria in Pvalb neurons likely affecting dendrite morphology.

Linked to the importance of Ca^2+^ signaling/homeostasis for neurotransmission in presynaptic terminals, mitochondria localized in presynaptic compartments affect Ca^2+^ signaling and neurosecretion [50–52], for more details on mitochondrial proteins, regulation, and cellular functions of mitochondrial Ca^2+^ (see [53]). One of the hypotheses—based on the common finding that ASD patient-derived cells (often neurons) show abnormalities in mitochondrial metabolism—places mitochondria dysfunction downstream of pathophysiological triggers, such as genetic mutations in ASD risk genes often affecting gene loci encoding Ca^2+^-signaling components leading to alterations in Ca^2+^ signaling [54, 55]. Mutations in ASD risk genes in animal models are associated with mitochondria dysfunction. Mitochondria isolated from different brain regions of Pten^+/-^mice (cerebellum, hippocampus, cortex) show age- and region-specific increases in the activities of mitochondrial complexes II–V, as well as of citrate synthase (the latter considered as a proxy measure for mitochondria mass), all suggestive of the increased mitochondria being optimally tuned for upholding ΔΨ_mito_ required for mitochondrial Ca^2+^ uptake [56]. Similarly, mitochondria from Fragile X Mental Retardation protein (FMRP)-deficient (*dfmr1*) *Drosophila* have increased maximum electron transport capacity (uncoupled from ATP synthase) [57]. In line, transcript encoding proteins implicated in mitochondrial Ca^2+^ uptake and generating ΔΨ_mito_, including mitochondrial calcium uptake 1 (*Micu1*), mitochondrial calcium uniporter regulator 1 (*Mcur1*), and cytochrome c oxidase subunit 1 (*COX1*) are elevated in PV^−/−^ kidney epithelial cells, a mechanism modifying the mitochondrial protein composition conceivably to increase the Ca^2+^-sequestration capacity of PV-deficient cells [58].

In addition, abnormalities in purine metabolism and purinergic signaling are present in *Fmr1* knockout mice. Blocking of ATP-mediated P2X and P2Y signaling by suramin corrects ASD-like features in these mice [59]. Disturbed redox homeostasis associated with oxidative stress (e.g., mitochondrial free radical overproduction) is often considered as a measure of mitochondria dysfunction and is observed in the ASD mouse model lacking functional methyl-CpG-binding protein 2, i.e., in Mecp2^−/−^ mice [60, 61]. Of note, redox alterations in Mecp2^−/−^ mice are transient, likely precluding irreversible neurodegeneration as the main mechanism implicated in ASD-associated oxidative stress [62]. Moreover, decreased levels of PV have been observed in Fmr1^−/−^ and Mecp2^−/−^ mice providing a link to the inverse change of mitochondria mass/activity and PV expression levels in PV-deficient mice (Table 1). In summary, studies in both human ASD and mouse models all indicate alterations in mitochondria protein/structure/function, yet a congruent picture on these changes with relation to ASD etiology/phenotype has not yet emerged.

**Table 1:** Animal ASD models with reported alterations in PV immunoreactivity, mitochondria dysfunction or hyper/hypo-connectivity

Protein / Gene Mouse (***animal***) model or Treatment	Altered PV staining/ PV^**+**^ number	Mitochondria dysfunction	Hyper/hypo-connectivty	SFARI^**a**^
Shank 3 (*Shank3*) Shank3B^-/-^	PV^+^ puncta surrounding pyramidal cells decreased in insular cortex of adult mice [1]^c^, PV downregulation in striatal Pvalb neurons at PND25 [2]	In a Fmr1 knock-in premutation mouse model resulting in Shank3 downregulation, at PND21, decreases in NADH oxidase, succinate oxidase and cytochrome *c* oxidase activity, as well as increased uncoupling between ATP production and electron transfer in hippocampus and cerebellum [3]	Altered local and global connectivity patterns indicative of circuit abnormalities in SHANK3-mutant ***macaques*** [4], prefrontal hypoconnectivity associated with reduced density of short-range cortical projections [5], reduced spine density in striatum of Shank3(Δex4–22)^-/-^ mice linked to abnormal functional connectivity within the cortico-striatal-thalamic circuit [6]	1 (S^b^)
phosphatase and tensin homolog (*Pten*) Pten^+/-^	n/a	Increase of several mitochondrial complex activities (II-III, IV and V) in mitochondria isolated from hippocampus and cerebellum (not cortex) of young (4-6 weeks) mice, not accompanied by increases in mitochondrial mass [7]	Increased axonal branching and connectivity (mPFC to basolateral amygdala axonal projections) [8], local and long-range hyper-connectivity in auditory cortex [9]	1 (S)
methyl CpG binding protein 2 (*Mecp2*) Mecp2^-/-^	At PND15 no PV^+^ cells [10] indicates delayed maturation of Pvalb neurons, but morphological hypermaturation in visual cortex is associated with increased *Pvalb* mRNA [11]	Increased ROS release in mitochondria isolated from hippocampus of Mecp2^-/-^ mice [12], increased H_2_O_2_ generation in mitochondria isolated from whole brain mainly produced by dysfunctional complex II [13]	Increase of Pvalb neuron cellular and PNN structural complexity in visual cortex [14], reduced density of excitatory dendritic spines in mPFC pyramidal cells [11]	2 (S)
contactin associated protein-like 2 (*Cntnap2*) Cntnap2^-/-^	Reduction in PV^+^ neurons in striatum and cortex at PND14 [15], PV downregulation in striatal Pvalb neurons at PND25 [16]	n/a	Decreased excitatory and inhibitory inputs onto mPFC L2/3 pyramidal neurons, concurrent with reduced spines and synapses [17], reduced long-range and local functional connectivity in prefrontal and midline brain "connectivity hubs" [18], major connectivity deficits in prefrontal and limbic pathways developing between adolescence and adulthood [19]	2 (S)
neuroligin 3 (*Nlgn3*) Nlgn3^R451C^	Asymmetric “patchy” PV-deficit in cortex at PND >60 [20]	n/a	Reduction in neuron firing synchrony in dissociated cultures of *rat* hippocampal neurons caused by a decrease in the complexity of axonal architecture [21]	2
fragile X mental retardation protein (*Fmr1*) Fmr1^-/-^	PV^+^ neurons reduced in somatosensory cortex layers II-VI in mice > 1 year [22]	Increased mitochondrial ROS production, impaired complex I activity, and increased mtDNA deletions in fibroblasts from *Fmr1* KI mice (described in [3]) [23], mitochondria isolated from *dfmr1*^*-/-*^***Drosophila*** thoraces show increased maximum electron transport system capacity under supersaturating conditions [24]	Anatomical hyperconnectivity in the primary visual cortex (V1), but a disproportional low connectivity of V1 with other neocortical regions [25], hyperconnectivity between neighbouring layer 5 pyramidal neurons during a critical period in early mPFC development [26], but robust hypoconnectivity phenotype in cortico-cortical and cortico-striatal circuits in PND30 mice [19]	3 (S)
Parvalbumin (*Pvalb*) PV^+/-^ and PV^-/-^	≈30% reduction of PV^+^ cells in PV^+/-^ mice in mPFC, SSC and striatum, no changes in numbers of Pvalb neurons in PV^+/-^ and PV^-/-^ mice at PND25 [2]	Increase in mitochondria volume and density in soma of Pvalb neurons and increased density and length of dendritic mitochondria in absence of PV expression [this study]	Increase in dendrite length (DG) and branching (striatum), as well as thickness of proximal dendrites (molecular layer interneurons) of selected PV^-/-^ Pvalb neurons (age 3 – 5 months) [this study]	5
Valproic acid (VPA) Treatment	Asymmetric PV deficit in cortex/hippocampus at PND >60 [20], PV downregulation in striatal Pvalb neurons at PND25 [27]	The antioxidant resveratrol shown to improve the *mitochondria* function of cells reverses decreases in gephryn expression observed in VPA-treated ***rats*** and restores the proportion of PV^+^ cells in the amygdala [28]	Increased synaptophysin immunostaining in mPFC and a synaptophysin deficit in all hippocampal subfields [29], enhancement of the local recurrent functional connectivity formed by neocortical pyramidal neurons, but diminished number of putative synaptic contacts in connections between layer 5 pyramidal neurons [30]	n/a

^a^Simons Foundation Autism Research Initiative (SFARI) gene scoring system (https://www.sfari.org/resource/sfari-gene/)

^b^*S* human syndromic

^c^References for data summarized in Table 1

[1] Gogolla, N., Takesian, A.E., Feng, G., Fagiolini, M. and Hensch, T.K. (2014). Sensory integration in mouse insular cortex reflects GABA circuit maturation. Neuron 83, 894-905.

[2] Filice, F., Vorckel, K.J., Sungur, A.O., Wöhr, M. and Schwaller, B. (2016). Reduction in parvalbumin expression not loss of the parvalbumin-expressing GABA interneuron subpopulation in genetic parvalbumin and shank mouse models of autism. Mol Brain 9, 10.

[3] Napoli, E. et al. (2016). Premutation in the Fragile X Mental Retardation 1 (*FMR1*) Gene Affects Maternal Zn-milk and Perinatal Brain Bioenergetics and Scaffolding. Front Neurosci 10, 159.

[4] Zhou, Y. et al. (2019). Atypical behaviour and connectivity in SHANK3-mutant macaques. Nature 570, 326-331.

[5] Pagani, M. et al. (2019). Deletion of Autism Risk Gene Shank3 Disrupts Prefrontal Connectivity. J Neurosci 39, 5299-5310.

[6] Wang, X. et al. (2016). Altered mGluR5-Homer scaffolds and corticostriatal connectivity in a Shank3 complete knockout model of autism. Nat Commun 7, 11459.

[7] Napoli, E. et al. (2012). Mitochondrial dysfunction in Pten haplo-insufficient mice with social deficits and repetitive behavior: interplay between Pten and p53. PLoS One 7, e42504.

[8] Huang, W.C., Chen, Y. and Page, D.T. (2016). Hyperconnectivity of prefrontal cortex to amygdala projections in a mouse model of macrocephaly/autism syndrome. Nat Commun 7, 13421.

[9] Xiong, Q., Oviedo, H.V., Trotman, L.C. and Zador, A.M. (2012). PTEN regulation of local and long-range connections in mouse auditory cortex. J Neurosci 32, 1643-52.

[10] Fukuda, T., Itoh, M., Ichikawa, T., Washiyama, K. and Goto, Y. (2005). Delayed maturation of neuronal architecture and synaptogenesis in cerebral cortex of Mecp2-deficient mice. J Neuropathol Exp Neurol 64, 537-44.

[11] Patrizi, A., Awad, P.N., Chattopadhyaya, B., Li, C., Di Cristo, G. and Fagiolini, M. (2019). Accelerated Hyper-Maturation of Parvalbumin Circuits in the Absence of MeCP2. Cereb Cortex doi: 10.1093/cercor/bhz085

[12] Can, K., Menzfeld, C., Rinne, L., Rehling, P., Kugler, S., Golubiani, G., Dudek, J. and Muller, M. (2019). Neuronal Redox-Imbalance in Rett Syndrome Affects Mitochondria as Well as Cytosol, and Is Accompanied by Intensified Mitochondrial O_2_ Consumption and ROS Release. Front Physiol 10, 479.

[13] De Filippis, B. et al. (2015). Mitochondrial free radical overproduction due to respiratory chain impairment in the brain of a mouse model of Rett syndrome: protective effect of CNF1. Free Radic Biol Med 83, 167-77.

[14] Sceniak, M.P., Lang, M., Enomoto, A.C., James Howell, C., Hermes, D.J. and Katz, D.M. (2016). Mechanisms of Functional Hypoconnectivity in the Medial Prefrontal Cortex of Mecp2 Null Mice. Cereb Cortex 26, 1938-1956.

[15] Penagarikano, O. et al. (2011). Absence of CNTNAP2 leads to epilepsy, neuronal migration abnormalities, and core autism-related deficits. Cell 147, 235-46.

[16] Lauber, E., Filice, F. and Schwaller, B. (2018). Dysregulation of Parvalbumin Expression in the Cntnap2-/- Mouse Model of Autism Spectrum Disorder. Front Mol Neurosci 11, 262.

[17] Lazaro, M.T. et al. (2019). Reduced Prefrontal Synaptic Connectivity and Disturbed Oscillatory Population Dynamics in the CNTNAP2 Model of Autism. Cell Rep 27, 2567-2578 e6.

[18] Liska, A. et al. (2018). Homozygous Loss of Autism-Risk Gene CNTNAP2 Results in Reduced Local and Long-Range Prefrontal Functional Connectivity. Cereb Cortex 28, 1141-1153.

[19] Zerbi, V. et al. (2018). Dysfunctional Autism Risk Genes Cause Circuit-Specific Connectivity Deficits With Distinct Developmental Trajectories. Cereb Cortex 28, 2495-2506.

[20] Gogolla, N., Leblanc, J.J., Quast, K.B., Sudhof, T.C., Fagiolini, M. and Hensch, T.K. (2009). Common circuit defect of excitatory-inhibitory balance in mouse models of autism. J Neurodev Disord 1, 172-81.

[21] Gutierrez, R.C., Hung, J., Zhang, Y., Kertesz, A.C., Espina, F.J. and Colicos, M.A. (2009). Altered synchrony and connectivity in neuronal networks expressing an autism-related mutation of neuroligin 3. Neuroscience 162, 208-21.

[22] Selby, L., Zhang, C. and Sun, Q.Q. (2007). Major defects in neocortical GABAergic inhibitory circuits in mice lacking the fragile X mental retardation protein. Neurosci Lett 412, 227-32.

[23] Song, G., Napoli, E., Wong, S., Hagerman, R., Liu, S., Tassone, F. and Giulivi, C. (2016). Altered redox mitochondrial biology in the neurodegenerative disorder fragile X-tremor/ataxia syndrome: use of antioxidants in precision medicine. Mol Med 22, 548-559.

[24] Weisz, E.D., Towheed, A., Monyak, R.E., Toth, M.S., Wallace, D.C. and Jongens, T.A. (2018). Loss of Drosophila FMRP leads to alterations in energy metabolism and mitochondrial function. Hum Mol Genet 27, 95-106.

[25] Haberl, M.G., Zerbi, V., Veltien, A., Ginger, M., Heerschap, A. and Frick, A. (2015). Structural-functional connectivity deficits of neocortical circuits in the Fmr1 (-/y) mouse model of autism. Sci Adv 1, e1500775.

[26] Testa-Silva, G., Loebel, A., Giugliano, M., de Kock, C.P., Mansvelder, H.D. and Meredith, R.M. (2012). Hyperconnectivity and slow synapses during early development of medial prefrontal cortex in a mouse model for mental retardation and autism. Cereb Cortex 22, 1333-42.

[27] Lauber, E., Filice, F. and Schwaller, B. (2016). Prenatal Valproate Exposure Differentially Affects Parvalbumin-Expressing Neurons and Related Circuits in the Cortex and Striatum of Mice. Front Mol Neurosci 9, 150.

[28] Fontes-Dutra, M. et al. (2018). Resveratrol Prevents Cellular and Behavioral Sensory Alterations in the Animal Model of Autism Induced by Valproic Acid. Front Synaptic Neurosci 10, 9.

[29] Codagnone, M.G., Podesta, M.F., Uccelli, N.A. and Reines, A. (2015). Differential Local Connectivity and Neuroinflammation Profiles in the Medial Prefrontal Cortex and Hippocampus in the Valproic Acid Rat Model of Autism. Dev Neurosci 37, 215-31.

[30] Rinaldi, T., Silberberg, G. and Markram, H. (2008). Hyperconnectivity of local neocortical microcircuitry induced by prenatal exposure to valproic acid. Cereb Cortex 18, 763-70.

### Reduced PV-induced mitochondria upregulation increased dendrite length and branching of Pvalb neurons: link to local hyperconnectivity in ASD

Yet another interesting link exists between PV levels, mitochondria volume, and neuropil/neuroglia branching. Mitochondria density/dynamics is a central determinant for branching of axons and dendrites during neurodevelopment [25]. Stalled, but metabolically active mitochondria along the axon, as well as mitochondria biogenesis are required for localized branch formation [40, 63]. Much less is currently known on the correlation between mitochondria and dendritic branching. Yet one may assume that branching of dendrites is an equally energetically demanding process requiring the presence of mitochondria in such microdomains. We have recently shown that PV overexpression in oligodendrocyte-like CG4 cells induces mitochondria downregulation in cell processes attenuating the processes’ lengthening and branching [28]. In this study with 3–5 months old (adult) PVKO-EGFP and PV-EGFP mice and in line with results obtained in young (PND18-24) PV^−/−^ mice [20], we found that dendritic branching of striatal Pvalb neurons remained increased in the absence of PV and thus generally accelerated maturation of PV^−/−^ networks is not a likely cause for the differences observed at PND18-24, but probably directly related to absence of PV. Moreover, the length of 2nd to 4th order branches of DG Pvalb neuron dendrites of PVKO-EGFP mice, as well as mitochondria length and density in DG, striatal, and MLI Pvalb neuron dendrites was increased indicating that dendrite mitochondria upregulation is a probable and possibly universal cause for increased processes’ length and branching in Pvalb neurons deficient for PV, a hypothesis to be examined in more detail in further studies. In general, augmented branching is linked to local hyperconnectivity, an effect previously reported in ASD children [64, 65] Of note, most human studies on connectivity changes in ASD were focused on projection neurons (see “Limitations”). In the case of the mouse ASD models Pten^+/−^ and Fmr1^−/−^, the alterations in connectivity were observed in the same strain, where increased mitochondria mass/function had been reported before (Table 1). Pten^+/−^ mice are characterized by larger outgrowth of dendrites in the auditory cortex [66], and increases are present in Fmr1^−/−^ [67], conditional Met receptor tyrosine kinase knockout (cKO-Met [68]; and Mdga2^+/−^ mice [69]). Exceptions are Cntnap2^−/−^ mice, where functional hypoconnectivity determined by fMRI was reported [70] and in the mPFC of Mecp2^−/−^mice, functional excitatory hypoconnectivity was postulated based on electrophysiological experiments [71] (Table 1). Of note, the absence of Mecp2 in mPFC, the brain region analyzed in this study, does neither affect inhibitory synaptic currents nor PV expression [71].

### High energy demand of Pvalb neurons, mitochondria dysfunction, and oxidative stress

Why is mitochondria dysfunction most strongly affecting Pvalb neurons? The prototypical Pvalb neurons, basket cells, axo-axonic (chandelier) cells in the hippocampus, and neocortex, have rather numerous unique features: fast-spiking properties, rapid action potential kinetics, perisomatic/axon initial segment inhibition of pyramidal cells, and high energy consumption (for details, see Table 1 in [72]). Because of the last, they contain considerably more mitochondria in their somata, dendrites, and axons than any other interneuron subpopulation or pyramidal cells (reviewed in [72]). Moreover, these mitochondria are enriched in proteins such as cytochrome c oxidases (complex IV) and cytochrome c, essential for mitochondrial electron transport generating ΔΨ_mito_. Of note, COX I, COX Vb, and cytochrome c are also strongly upregulated in fast-twitch muscle of PV^−/−^ mice [38]. The high energy requirement is also likely linked to the fact that Pvalb neurons and their synchronized firing is a prerequisite for the emergence of gamma oscillations in hippocampus [73] and neocortex [8], a network function temporally binding neuron ensembles into a common regime during e.g., sensory perception and motor, as well as social behavior [72]. During such oscillations, mitochondria are close to their limit of mitochondrial oxidative capacity (reviewed in [72]). Conditional knockout of *Cox10* in Pvalb neurons elicits an energy deficit in those neurons that leads to circuit dysfunction, impaired sensory gating, and of importance in view of a link to ASD, to social disability evidenced in the 3-chamber sociability task [74]. High energy consumption and upholding a large ΔΨ_mito_ in Pvalb neurons evidently also increases ROS production. Although physiological levels of mitochondrial ROS regulate many cellular processes [75], elevated ROS levels lead to oxidative stress and impairment of neuron function reported in ASD [21] and schizophrenia [76], in the latter also with the involvement of Pvalb neurons [77]. In line with a protective effect of PV, a reduction of ROS production is seen in PV-overexpressing CG4 cells in vitro [28]. Preliminary data in PV^−/−^ mice supports the hypothesis that absence of PV resulting in mitochondria volume increase also augments ROS production in Pvalb neurons in a PV concentration-dependent and age-dependent way (Janickova and Schwaller, in preparation).

### Limitations

As for all animal models relating to a human (neurodevelopmental) disease, species differences have to be taken into account. While the distribution and also the developmental trajectory of Pvalb neurons in various brain regions have been extensively studied in the rodent (rat, mouse) brain [5, 9, 78], much less is known on Pvalb neurons in the human brain [6, 79, 80]. Based on the lower density of Pvalb neurons in the human neocortex [6] compared to mice and subsequently probably different connectivity of Pvalb neurons with other cortical neurons (interneurons, pyramidal cells), the downregulation of PV (or Pvalb neuron loss) observed in the neocortex in human ASD [6, 79] is likely to differently affect network dynamics and (morphological) alterations of brain circuits. Thus, until demonstrated in human ASD brains, the reported changes on mitochondria volume and dendritic branching of Pvalb neurons caused by PV downregulation are restricted to the mouse brain. In this study, we do not provide experimental evidence that increased length and branching of dendrites in PV^−/−^ Pvalb neurons directly translates into increased number of synaptic inputs onto Pvalb neurons (excitatory and/or inhibitory) leading to functional hyperconnectivity. Such a claim needs to be substantiated by detailed EM studies of Pvalb neuron morphology and connectivity, as well as by electrophysiological experiments specifically aimed to address this question. Although hyperconnectivity and mitochondria dysfunction has been reported in several ASD mouse models (Table 1), the direct link to PV downregulation has to be determined experimentally in those models. Moreover, our analyses are restricted to inhibitory Pvalb neurons, while most studies on changes in human ASD brain connectivity were focused on projection neurons, e.g., with respect to fine structure of axons [81] (for more details, see [4]) or dendritic spine density [82].

## Conclusions

Many of the pathophysiological features previously reported in ASD are observed in mice deficient for PV: altered Ca^2+^ signals, i.e., a lower initial rate in [Ca^2+^]_i_ decay in Pvalb neurons including Purkinje cells, stellate, and basket cells, increased short-term facilitation affecting E/I balance, augmented mitochondria volume possibly associated with increased oxidative stress and finally increased neurite (dendrite) branching likely augmenting local network connectivity (for more details, see chapter on PV function in [15]). Most of these features are also seen in several other genetic or environmental (e.g., VPA) mouse ASD models, where a reduction of PV levels (often reported as “decrease in PV-positive neurons”) was observed (Table 1). Thus, further investigations on alterations in PV expression levels, mitochondria dysfunction and neuron connectivity are highly warranted in the existing (and novel) mouse genetic ASD models, aimed at elucidating, whether changes reported in this study are common pathophysio-morphological alterations in ASD and thus PV-deficiency may represent a point of convergence in ASD etiology.

## Supplementary information

**Additional file 1:** Supplementary materials

**Additional file 2.** Movie 1 (AVI 17404 kb)

**Additional file 3.** Movie 2

**Additional file 4.** Movie 3

## Data Availability

The datasets used and/or analyzed during the current study are available from the corresponding author on reasonable request.

## Abbreviations

[Ca^2+^]_i_: Intracellular calcium concentration
ASD: Autism spectrum disorder
CA1: Cornu ammonis 1
Ca^2+^: Calcium ions
CA3: Cornu ammonis 3
CaBP: Ca^2+^-binding protein
COX: Cytochrome oxidase
DG: Dentate gyrus
E/I: Excitation/inhibition
EGFP: Enhanced green fluorescent protein
ETC: Electron transport chain
GABA: γ-aminobutyric acid
IHC: Immunohistochemistry
MLI: Molecular layer interneurons
mPFC: Medial prefrontal cortex
PND: Postnatal day
PV: Parvalbumin
ROS: Reactive oxygen species
SSC: Somatosensory cortex
TBS: Tris-buffered saline
TRN: Thalamic reticular nucleus
VPA: Valproic acid
WT: Wild-type
