# Correction to: Absence of parvalbumin increases mitochondria volume and branching of dendrites in inhibitory Pvalb neurons in vivo: a point of convergence of autism spectrum disorder (ASD) risk gene phenotypes

**DOI:** 10.1186/s13229-020-00404-8

**Published:** 2021-02-05

**Authors:** Lucia Janickova, Karin Farah Rechberger, Lucas Wey, Beat Schwaller

**Affiliations:** Anatomy, Section of Medicine, University of Fribourg, Route Albert-Gockel 1, 1700 Fribourg, Switzerland

## Correction to: Janickova et al. Molecular Autism (2020) 11:47 10.1186/s13229-020-00323-8

Following publication of the original article [1], the authors identified an error that occurred during publication process and Fig. 7 was not published. Figure 7 is provided below:

**Fig. 7:**
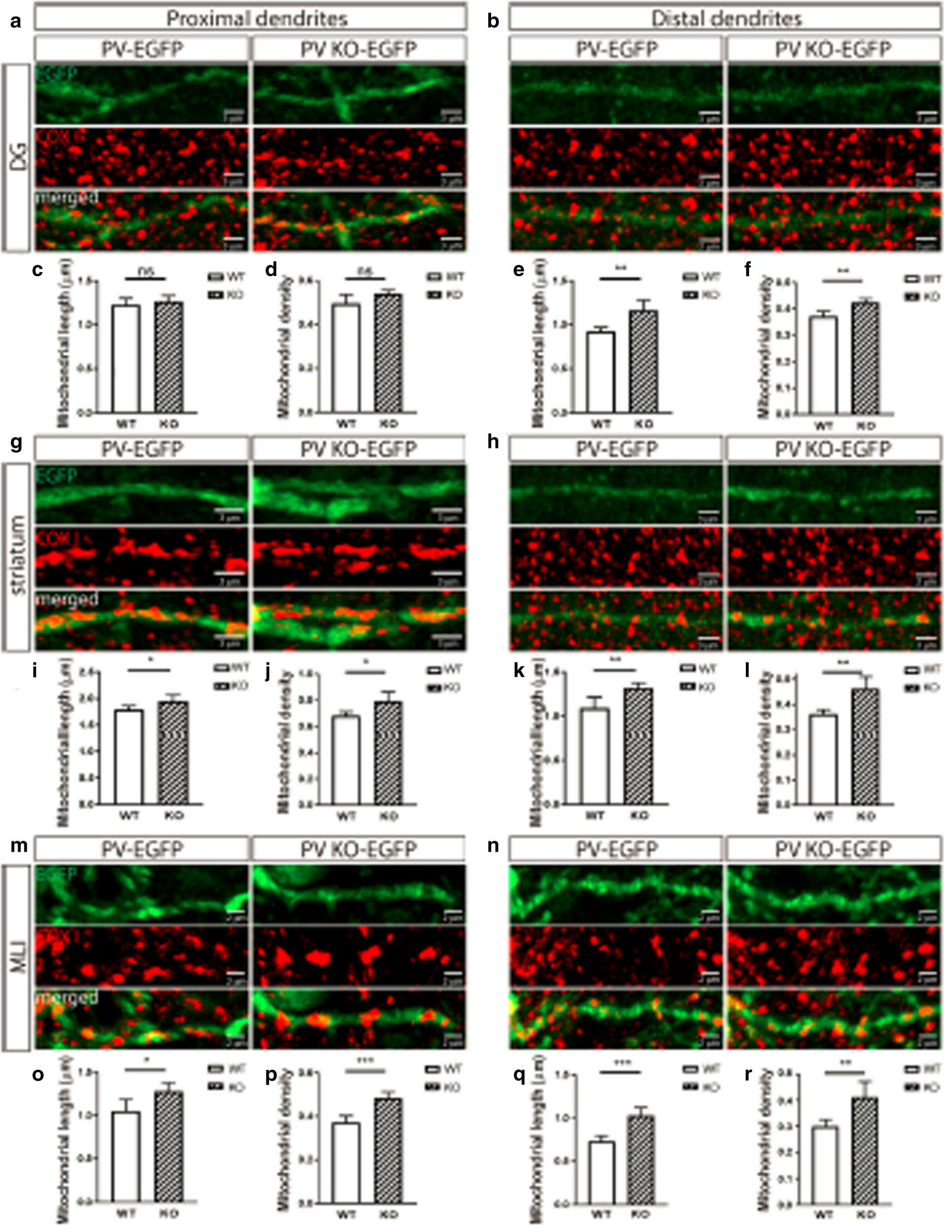
Mitochondria length and density in proximal and distal dendrites of hippocampal DG (**a**–**f**), striatal (**g**–**l**), and MLI (**m**–**s**) Pvalb neurons from PV-EGFP (WT) and PVKO-EGFP (KO) mice. **a** Representative images of proximal dendrites from DG Pvalb neurons of a PV-EGFP (left) and a PVKO-EGFP (right) mouse showing the overall dendrite morphology (EGFP, top), mitochondria (COX I, middle), and the merged image (bottom). **b** Images from distal dendrites (as in (**a**)). Average length and density of mitochondria in proximal (**c**, **d**) and distal (**e**, **f**) dendrites of DG Pvalb neurons. Representative images (**g**, **h**) and quantitative analyses (**i**–**l**) from striatal Pvalb neurons. Representative images (**m**, **n**) and quantitative analyses (**o**–**s**) from MLI Pvalb neurons. For all graphs showing quantitative data: n = 10 randomly selected cells and ns: not significant, *p < 0.05, **p < 0.01, ***p < 0.001

The publisher apologizes to the authors and readers for the error and inconvenience.
