# Supplementary material for: Absence of parvalbumin increases mitochondria volume and branching of dendrites in inhibitory Pvalb neurons in vivo: a point of convergence of autism spectrum disorder (ASD) risk gene phenotypes
Source: Mol Autism. 2020 Jun 9;11:47. doi: 10.1186/s13229-020-00323-8 (PMC7285523; doi:10.1186/s13229-020-00323-8)
Supplement: Supplementary file 1 — Additional file 1: Supplementary materials [file 13229_2020_323_MOESM1_ESM.pdf]

Legends to Supplemental Figures

Supplemental Figure S1 Pvalb neuron selection criteria for quantitative analysis.

Nanozoomer images (coronal sections) from a PV-EGFP mouse are shown in brightfield **(A)** and fluorescence **(B)** mode; scale bar 1 mm. Pvalb neurons were identified based on EGFP immunolabeling (green). DAPI staining (blue) helped to navigate through the selected brain regions. For analyses, brain sections taken at specific distances from bregma (marked above the images) were first scanned with the Nanozoomer to visualize the entire brain sections in a macroscopic view **(A, B)**. The same sections were subsequently scanned by confocal microscopy **(C)** in regions of interest (mPFC, striatum, CA1, CA3, DG, SSC, TRN, cerebellum) at higher magnification; areas (390 x 390 µm) marked by white squares in **(B)** are shown in **(C)**. Confocal images are shown as z-stack maximal intensity projections (390 x 390 x 40 µm). Scale bars: 30 µm. Pvalb neurons identified as $EGFP^{+}$ cells were selected from the z-stack of images, only if cell somata were entirely within the stack (not cropped in the x-y plane nor along the z-axis; neurons fulfilling the criteria were marked by numbers. Numbered cells were used for quantitative analyses.

Supplemental Figure S2 Absence of striking differences in overall brain morphology observed in sagittal sections of PV-EGFP and PVKO-EGFP mice (A-C) and surface estimation of various brain regions obtained from whole brain dorsal view images (D) and Cresyl violet-stained sagittal sections (E) from PV-EGFP and PVKO-EGFP mice. A) Nanozoomer images (sagittal sections) from a PV-EGFP (top) and a PVKO-EGFP mouse (bottom) stained for EGFP (green) and DAPI (blue); scale bar 1 mm. B) Higher magnification confocal images of the cerebellum from the sections shown in A). Note complete absence of PV (red staining) in the PVKO-EGFP mouse. The merged images show a complete overlap (yellow) in PC and MLI of a PV-EGFP mouse. Scale bar: 20 µm. C) Left part: Western blot from cerebellar extracts from the 2 genotypes show a band for GAPDH (control) and EGFP in both extracts. A band for PV is present only in the PV-EGFP sample. Right part: Ponceau red staining of the

membrane reveals equal loading of the 2 samples. Molecular weight markers are shown in the left lane. D) Representative dorsal view images from a PV-EGFP (top) and a PVKO-EGFP (bottom) mouse. No differences in surfaces of olfactory bulbs, cortex, colliculi cerebellum and whole brain were observed between WT (solid red circles) and KO (open red circles); n = 5 mice per genotype. E) On sagittal sections, no differences in the surface area of hippocampus, cortex, striatum, cerebellum and thalamus were detected between WT and KO mice; n = 5 mice per genotype. Scale bar: 1 mm.

Supplemental Figure S3 Increase in mitochondrial volume (B) and density (C) in Pvalb neurons from PVKO-EGFP mice exceeds increase in total cell (soma) volume (D) and highly correlates with estimated PV concentrations (A). The % increase in mitochondria volume (B) and moreover density (C) compared to PV-EGFP (WT) is strongly correlated with the increase in overall soma volume (D). Of note the relative increase in mitochondria volume and density is generally larger than the effect of PV on the increase in soma volume (compare the scale on the y-axis of B) and C) with D)). However, note that all parameters correlate with the PV concentration estimated for the various Pvalb neuron subpopulations (A). The higher the concentration of PV, the larger is the increase in mitochondria density in the absence of PV, i.e. in Pvalb neurons of PVKO-EGFP mice. The only exception is the Purkinje cell population, where no increase in cell soma volume is observed in absence of PV likely due to topological constraints (see discussion for details). Moreover, the increase in mitochondria density is overproportional in striatal Pvalb neurons (C) characterized by a relatively low PV concentration. Thus, other parameters, e.g. firing properties and/or neuronal activity of Pvalb neurons might be correlated with PV levels, absolute mitochondria volume and mitochondria density in a given Pvalb neuron subpopulation.

Supplemental Figure S4 Relative (%) increase in length (A,C) and density (B,D) of mitochondria in proximal (A,B) and distal (C,D) dendrites of PVKO-EGFP mice compared to PV-EGFP mice. Changes were calculated for Pvalb neurons in DG, striatum and cerebellum (MLI) (n = 10 neurons per region and genotype). Note the similar changes with respect to mitochondria length and density in proximal and distal dendrites and the correlation with the PV concentrations (suppl. Fig. 3A) in the three Pvalb neuron subpopulations.

Legends to supplemental movies

Movie M1

3D image (z-stack) from a striatal $EGFP^+$ Pvalb neuron from a PV-EGFP (WT) mouse. Absence of green dendrites from other Pvalb neurons allows for automated reconstruction and quantification of dendritic branching as shown in Fig. 6D,E,F.

Movie M2

Example of a proximal dendritic segment (approximately 30 µm; green) of a striatal Pvalb neuron similar to the ones used for quantification of mitochondria length and mitochondria density shown in Fig. 7. From the soma (left) a proximal dendrite segment is extending nearly perpendicular to the z-axis, i.e. within the x-y plane. Reconstructed mitochondria are shown in red. Then, green and red images are merged. The reconstructed dendrite volume (‘surface mode’ set to transparent) allows to identify mitochondria within the segment. For visualization, the the z-axis is rotated by 360°.

Movie M3

TRN Pvalb neurons are flat ellipsoid (discus-like) structures. The cytoplasm transparent mask (green) contains mitochondria (red) and a nucleus (blue).

Supplemental Material

Since the fluorescence intensity values were obtained from maximal density projections (z-stacks) and the morphology of the various Pvalb neuron subpopulations are quite different, a correction factor based on Pvalb neuron morphology was calculated. Assuming that neurons and nuclei can be approximated by spheres, from the volume of the entire soma and the nucleus, the radii of these 2 spheres (r2: soma; r1: nucleus) were calculated (suppl. Table S1). The difference in the radii is then the thickness (d) of the shell comprising the cytoplasmic volume (see below).

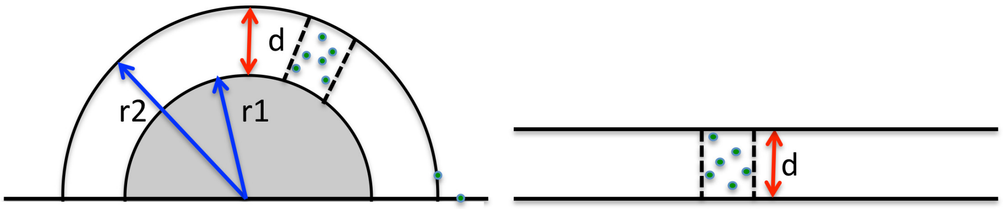

The number of EGFP molecules (green dots) present in a shell segment is assumed to be approximately proportional to d. Thus, average fluorescence intensity values for each Pvalb neuron subpopulation were divided by d resulting in corrected fluorescence values. From the standard curve using the reported PV concentrations for hippocampal Pvalb neurons (~20 µM), Purkinje cells (~100 µM) and MLI (~570 µM), the PV concentration of striatal and cortical, as well as TRN Pvalb neurons was calculated. In case of TRN neurons an additional correction was introduced. While TRN Pvalb neurons appear roundish in coronal sections (Fig. 5C), in horizontal and sagittal sections they are seen as flat elliptic structures, thus the overall shape of the TRN Pvalb neurons is a thin nearly disc-shaped ellipsoid lying in the coronal plain, i.e. the short axis of the ellipsoid oriented rostral-caudal (see also Fig. 5 in (70) and suppl. Movie 3. To account for this ellipsoid shape we chose d/2 (blue in suppl. Table S1) as the thickness of the shell for TRN Pvalb neurons, since coronal sections were used for analysis. This resulted then in a corrected value for the EGFP signal intensity (marked in blue). We conjecture that this factor results likely in an underestimation of the "true" PV concentration. A graph showing corrected EGFP fluorescence values (x-axis) *vs.* reported PV concentrations (y-axis) for hippocampal Pvalb neurons, Purkinje cells and cerebellar basket cells (red values in suppl. Table S1) yields a linear

relationship (Fig. 1D). Using this calibration curve, plotting of the corrected fluorescence values for the other Pvalb neuron subpopulations resulted in estimates for the PV concentration in the 4 regions mPFC, SSC, striatum and TRN (Fig. 1E and suppl. Table S1).

Supplemental Table S1

Brain region	Volume soma (μm^3)	Radius (r2) soma (µm)	Volume nucleus (μm^3)	Radius (r1) nucleus (µm)	Thickness shell (d) (µm)	EGFP signal (a.u.)	Corr. EGFP signal	PV conc. (µM)*
CA1	1517.6	7.13	461.0	4.79	2.34			
CA3	1672.8	7.36	539.0	5.05	2.32	55	24.6	20
DG	1547.2	7.17	559.6	5.11	2.06			
striatum	1147.6	6.49	323.8	4.26	2.24	72	32.2	~70
SSC	1163.8	6.53	284.4	4.08	2.45	83	33.9	~90
mPFC	970.6	6.14	236.6	3.84	2.31	88	38.2	~125
TRN	738.4	5.61	210.4	3.69	1.92 (0.96)**	139	145.0	~750
PC	2914.0	8.86	668.4	5.42	3.44	129	37.5	100
MLI	250.0	3.91	85.2	2.73	1.18	119	101.0	570

The standard curve established from the values in red (obtained from a sagittal section of one mouse) resulted in the following regression line:

$y = 0.1376x + 22.721$; $R^2 = 0.99942$

* Values are the average from 2 mice (10 – 15 cells/mouse/brain region), i.e. obtained from 2 standard curves with different regression lines.

** Blue values for TRN (d/2) are based on the morphology of TRN neurons (for details, see text « Supplemental Material » and movie M3.

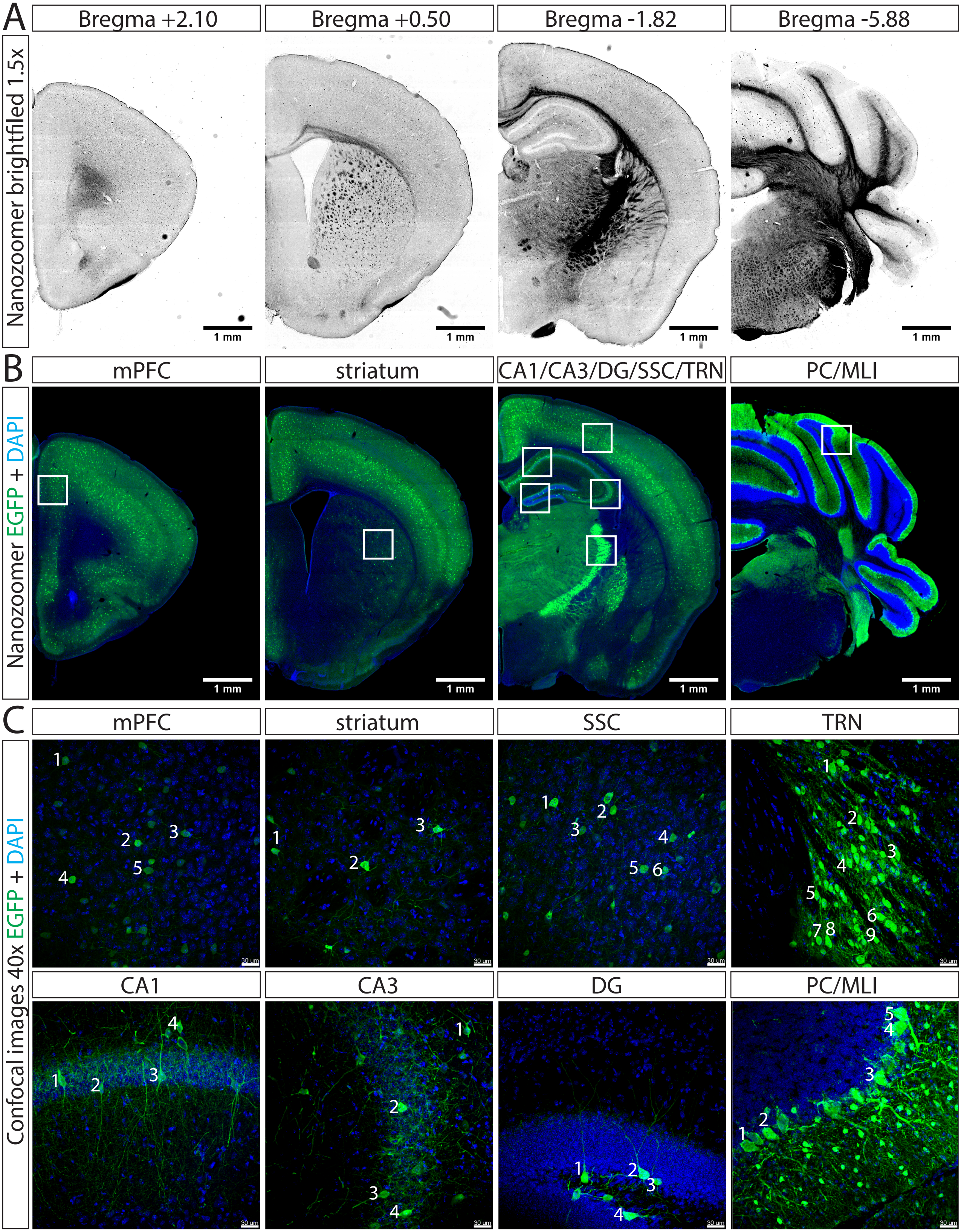

A
Bregma +2.10
Bregma +0.50
Bregma -1.82
Bregma -5.88
Nanozoomer brightfiled 1.5x
1 mm
B
mPFC
striatum
CA1/CA3/DG/SSC/TRN
PC/MLI
Nanozoomer EGFP + DAPI
C
mPFC
striatum
SSC
TRN
CA1
CA3
DG
PC/MLI
Confocal images 40x EGFP + DAPI
30 um

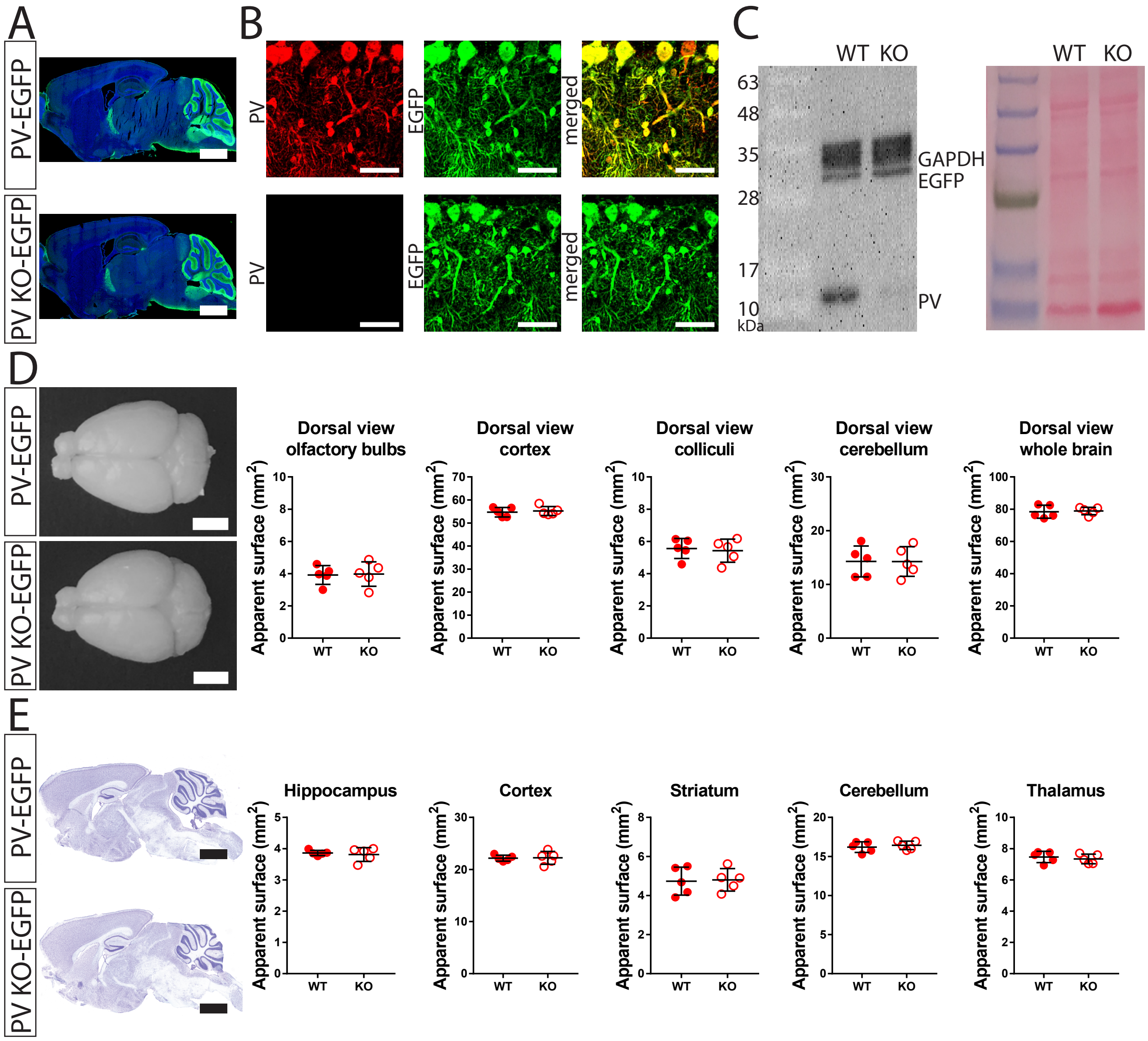
A
PV-EGFP
PV KO-EGFP
B
PV
EGFP
merged
C
WT KO
63
48
35
28
17
10
kDa
GAPDH
EGFP
PV
D
Dorsal view olfactory bulbs
Dorsal view cortex
Dorsal view colliculi
Dorsal view cerebellum
Dorsal view whole brain
Apparent surface (mm2)
WT
KO
E
Hippocampus
Cortex
Striatum
Cerebellum
Thalamus

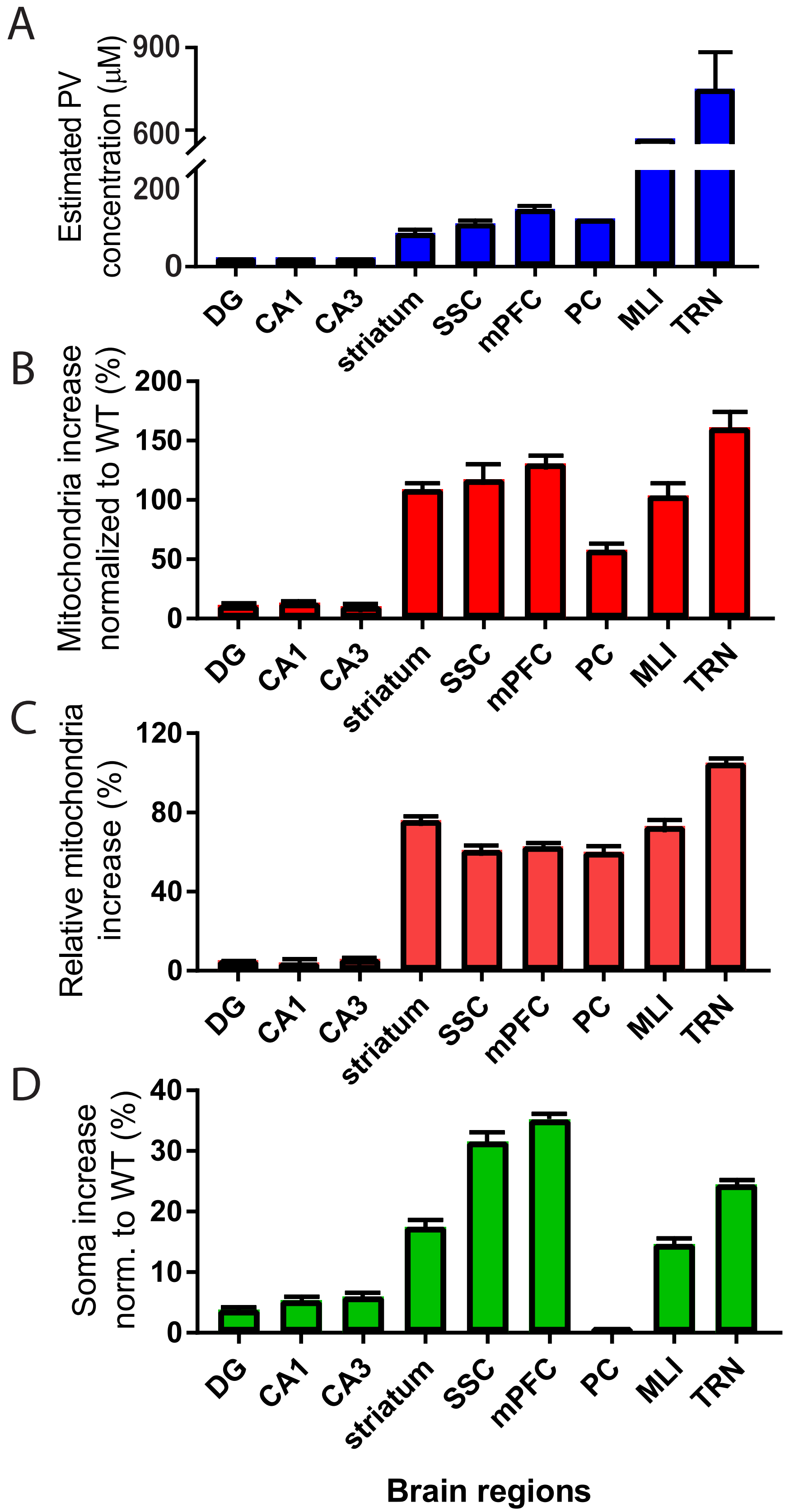

A
Estimated PV concentration (μM)
900
600
200
0
DG
CA1
CA3
striatum
SSC
mPFC
PC
MLI
TRN
B
Mitochondria increase normalized to WT (%)
200
150
100
50
0
C
Relative mitochondria increase (%)
120
80
40
0
D
Soma increase norm. to WT (%)
40
30
20
10
0
Brain regions

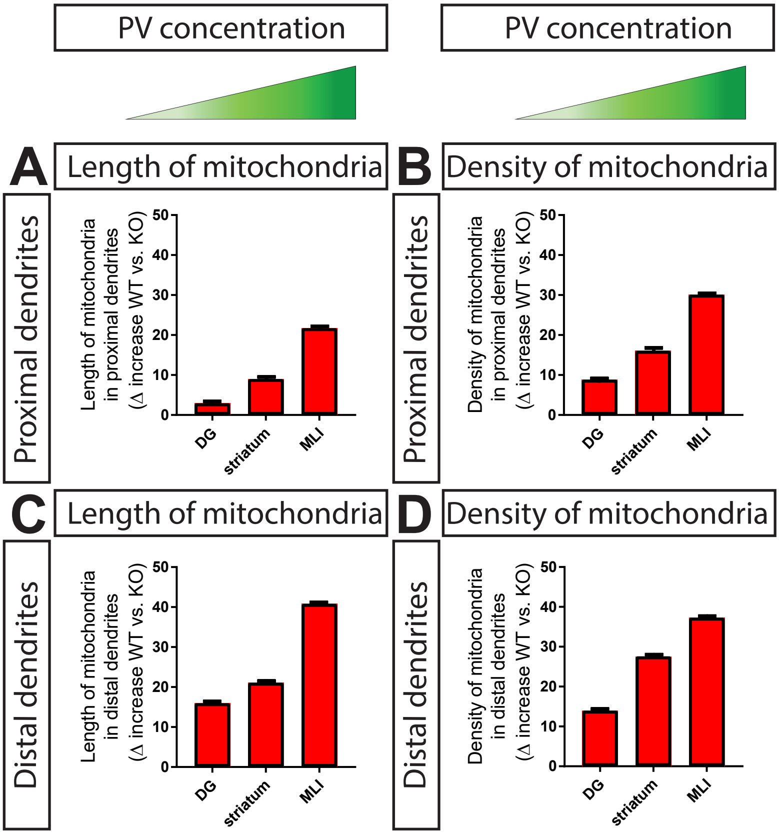
PV concentration
PV concentration
A
Length of mitochondria
B
Density of mitochondria
Proximal dendrites
Length of mitochondria in proximal dendrites (Δ increase WT vs. KO)
Proximal dendrites
Density of mitochondria in proximal dendrites (Δ increase WT vs. KO)
C
Length of mitochondria
D
Density of mitochondria
Distal dendrites
Length of mitochondria in distal dendrites (Δ increase WT vs. KO)
Distal dendrites
Density of mitochondria in distal dendrites (Δ increase WT vs. KO)
0
10
20
30
40
50
DG
striatum
MLI
